# Polystyrene-degrading bacteria modulate host stress and toxicity responses to microplastic exposure in *Caenorhabditis elegans*

**DOI:** 10.1093/ismejo/wrag051

**Published:** 2026-03-10

**Authors:** Min-Geun Kang, Daniel Junpyo Lee, Arthur Junghun Kim, Ki Beom Jang, Anna Kang, Youbin Choi, Jihyun Yoon, Eunsol Seo, Younghoon Kim

**Affiliations:** Department of Agricultural Biotechnology and Research Institute of Agriculture and Life Science, Seoul National University, Seoul 08826, Republic of Korea; Department of Agricultural Biotechnology and Research Institute of Agriculture and Life Science, Seoul National University, Seoul 08826, Republic of Korea; Department of Agricultural Biotechnology and Research Institute of Agriculture and Life Science, Seoul National University, Seoul 08826, Republic of Korea; Department of Agricultural Biotechnology and Research Institute of Agriculture and Life Science, Seoul National University, Seoul 08826, Republic of Korea; Department of Agricultural Biotechnology and Research Institute of Agriculture and Life Science, Seoul National University, Seoul 08826, Republic of Korea; Department of Agricultural Biotechnology and Research Institute of Agriculture and Life Science, Seoul National University, Seoul 08826, Republic of Korea; Department of Agricultural Biotechnology and Research Institute of Agriculture and Life Science, Seoul National University, Seoul 08826, Republic of Korea; Department of Agricultural Biotechnology and Research Institute of Agriculture and Life Science, Seoul National University, Seoul 08826, Republic of Korea; Department of Agricultural Biotechnology and Research Institute of Agriculture and Life Science, Seoul National University, Seoul 08826, Republic of Korea

**Keywords:** polystyrene microplastic, microplastic toxicity modulation, host-bacteria-microplastic interactions

## Abstract

Microplastic exposure is an emerging health risk. Host-associated plastic-degrading commensal bacteria can directly interact with microplastic particles and alter them physically and chemically, thereby potentially modulating microplastic toxicity. Despite numerous reports of plastic-degrading bacteria isolated from host intestines, how these interactions affect host physiology remains unclear. Here, we compared two polystyrene-degrading bacteria—Enterobacter hormaechei LG3 and Bacillus amyloliquefaciens SCGB1—in Caenorhabditis elegans exposed to laboratory-manufactured 1-μm polystyrene microspheres (Mi-PS). LG3-fed worms showed dose-dependent physiological impairment in response to Mi-PS, whereas SCGB1-fed worms exhibited attenuated or negligible impairment. The strains interacted with Mi-PS via distinct physiological and metabolic responses, reflected by differences in biofilm formation, particle attachment, and metabolite profiles. These strain-specific differences were confirmed to directly influence host outcomes. Under identical exposure conditions (10 mg/L, 50 h), LG3-fed worms accumulated more Mi-PS particles in the gut than SCGB1-fed worms (n = 48; mean ± SD, 3.28 ± 4.22 vs 0.63 ± 1.03 particles per worm). A transcriptome-guided validation framework provided mechanistic clues to strain-specific microplastic interactions. LG3-associated impairment coincided with the formation of oxidized Mi-PS particles, production of oxidized styrene intermediates, and microparticle-driven changes in bacterial cell properties, including activation of the lipopolysaccharide biosynthesis pathway. In contrast, SCGB1-associated attenuation was consistent with isobutyrate/isovalerate-mediated modulation of host DAF signaling. Collectively, these results link bacteria–microplastic interactions to host outcomes and offer actionable insight for assessing and mitigating microplastic-related health risks.

## Graphical Abstract

**Graphical Abstract:**
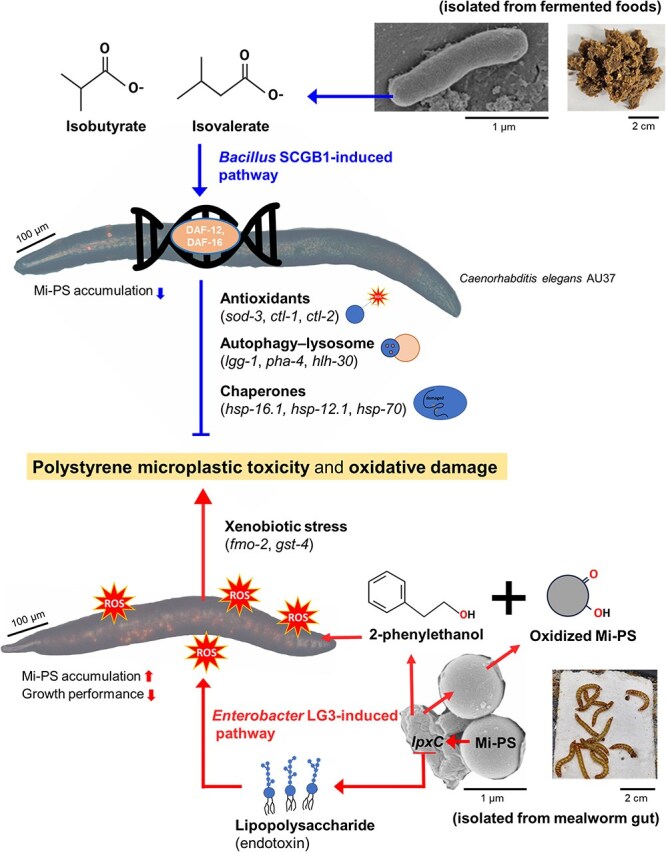

## Introduction

Synthetic plastics have established an irreplaceable industrial position because they are durable, lightweight, and inexpensive, and their global production exceeded 400 million metric tons in 2023 [1–3]. This growth has driven the accumulation of plastic waste and widespread environmental exposure to microplastics [4]. Polyethylene, polypropylene, and polystyrene (PS) are among the most common polymer types detected in environmental microplastics [5], and PS-based polymers have been assigned relatively high hazard scores [6]. Expanded polystyrene (EPS), a foamed form of PS with entrapped air, has low density, high buoyancy, thermal insulation, and shock-absorbing capacity [7]. These properties have supported broad use of EPS in the built environment, marine aquaculture, and food packaging, including instant noodle containers and insulated boxes [7–9]. However, EPS fragments readily; because PS is chemically stable and resists biodegradation, fragmented EPS can persist and contribute to bioaccumulative polystyrene microplastics (PS-MPs) [10–14]. Consistent with this exposure potential, PS-MPs have been detected in wild-caught aquatic organisms and have also been reported in human fecal samples, indicating that unintentional exposure can occur through environmental and consumer-product pathways [15–17].

Bioaccumulated PS-MPs disrupt biological systems across taxa. In Caenorhabditis elegans, chronic PS-MPs exposure induces oxidative stress, impairs locomotion, and shortens lifespan [18]. Similar disturbances in digestive and neural functions have been reported in vertebrates, where PS-MPs disrupt microbiota, elevate lipopolysaccharide (LPS) levels, and induce neuroinflammation [19]. These observations motivate a mechanistic understanding of when and why toxicity manifests—and whether it can be mitigated.

Several polystyrene-degrading bacteria isolated from animal hosts can biodegrade PS-MPs by adhering to their surfaces and catalyzing enzymatic oxidation and depolymerization [20–22]. Such microorganisms and PS-MPs interactions could remodel the physicochemical properties of PS-MPs and generate diverse metabolites from PS-MPs, thereby altering gut retention and host responses. Despite numerous reports identifying plastic-degrading bacterial strains, the in vivo outcomes of co-exposure to plastic-degrading bacteria and microplastics remain poorly understood [23, 24].

We previously identified two PS-degrading strains, Enterobacter hormaechei LG3 and Bacillus amyloliquefaciens SCGB1, which follow distinct degradation routes and produce different PS-derived metabolites under carbon-limited conditions [21]. When PS-MPs served as the sole carbon source, LG3 upregulated LPS biosynthetic genes (*lpxA*, *lpxC*, *lpxD*) and biofilm-associated loci, suggesting that PS catabolism can couple to bacterial immunogenic pathways [25]. Clarifying how these biotransformed PS-MPs, together with the remodeled microbial physiological activity induced by PS exposure, ultimately affect the host is essential, because such insights could reveal a previously underappreciated dimension of host–bacteria–microplastic interactions [26].

Here, we used C*.* elegans and laboratory-manufactured 1-μm polystyrene microspheres (Mi-PS) as an in vivo model system for PS-MP exposure. Two PS-degrading strains (LG3 and SCGB1) were compared with Escherichia coli OP50, the standard laboratory food for C. elegans, which did not exhibit PS-degrading activity. We addressed three linked questions: (i) How do interactions between Mi-PS and PS-degrading bacteria alter the properties of each component? (ii) How do these microplastic–bacteria interactions translate into changes in host physiology? (iii) Which microbially induced host signaling axes play key roles in modulating Mi-PS toxicity?

Bacterial strain choice was motivated by contrasting ecological and physiological roles. E*.* hormaechei is a facultative anaerobe commonly found in animal intestines and displays context-dependent host interactions—it can modulate immune responses or impair host health through pathogenic outcomes, and several isolates are capable of biodegrading polystyrene [27–30]. In line with this duality, we examined whether strain LG3 undergoes cellular alterations—particularly in lipopolysaccharide (LPS) biosynthesis—during exposure with Mi-PS. By contrast, B*.* amyloliquefaciens is associated with intestinal homeostasis and mitigation of oxidative stress, and several lines biodegrade PS [21, 31–34]. These differences provide a tractable framework for testing whether microbiome composition can affect microplastic risk.

C. elegans provides a transparent, genetically tractable in vivo platform for real-time assessment of microplastic uptake and toxicity. Because C. elegans is a short-lived bacterivore, it enables rapid readouts of survival, oxidative stress, and behavioral endpoints under defined bacterial diets [35–37]. The C. elegans AU37 strain was primarily used; in this strain, p38 MAPK signaling is compromised, rendering animals hypersensitive to pathogenic, xenobiotic, and oxidative stress [38].

We co-exposed C. elegans to Mi-PS with LG3, SCGB1, or OP50 and assessed host physiological outcomes. These results show that the magnitude of Mi-PS toxicity depends strongly on strain identity: LG3 increases Mi-PS bioaccumulation and oxidative stress, whereas SCGB1 mitigates both. Strain-specific responses to Mi-PS and host transcriptomes were analyzed to outline a host–bacteria–microplastic pathway where Mi-PS alters bacterial metabolism and physiology, bacteria modify particle properties, and both together determine host susceptibility. These findings indicate that specific bacterial populations can critically modulate microplastic toxicity and point to microbiome management as an actionable lever to mitigate associated risks.

## Materials and methods

### Key reagents and resources

The detailed description of the reagents and resources used is presented in Table S1.

### 1-μm and 25-μm polystyrene microspheres and silica microspheres

Microparticles with a diameter of 1 μm were used for C. elegans exposure. Fluorescent 1-μm Mi-PS internally labeled with polychromatic red dye were used as a model PS-MP. The dye was embedded in the particle core rather than surface-coated (manufacturer specification). Particle morphology and fluorescence were verified (Fig. S1A–C). To confirm that labeling did not alter surface chemistry, FTIR spectra of Mi-PS and a non-labeled PS film were compared and showed no dye-specific peaks (Fig. S1D). The molecular weight (M_w_) of Mi-PS was 2.77 × 10^5^ Da (Fig. S1E). For a non-polymeric particle control, 1-μm Mi-Si were used. Their comparable specifications (including particle size and morphology) were verified (Fig. S2).

Microparticles with a diameter of 25 μm were used to generate attached biofilms and to harvest biofilm-induced biomass after removing the microparticles (Fig. S3). The protocol for preparing biofilm-induced biomass is described in Text S1.

### Caenorhabditis elegans

Nematodes were maintained on nematode growth medium (NGM) agar plates seeded with E. coli OP50 at 20°C [39]. All experimental strains were obtained from the Caenorhabditis Genetics Center (CGC) and are listed in Table S1. Synchronized L1 populations were generated using a standard alkaline hypochlorite bleaching protocol [39]. Synchronized L1 larvae were used for experiments at 25°C under oxic conditions.

### M9 buffer and modified nematode growth medium

M9 buffer and modified NGM were prepared by standard recipes [39, 40]. Antibiotic variants (ampicillin/streptomycin, 100 μg/ ml each) were used where suppression of bacterial growth was required [41]. Compositions, sterilization steps, and storage conditions are provided in Text S2.

### Bacterial strains and preparation

Three strains were used: *E. coli* OP50 (PS-degradation-negative control), E. hormaechei *LG3* (isolated from the gut of Tenebrio molitor), and B. amyloliquefaciens SCGB1 (isolated from the fermented food cheonggukjang). Prior work established PS-degradation by LG3 and SCGB1 under aerobic conditions [21]. Here, OP50’s negative status was confirmed on carbon-free basal medium with PS substrates (no growth or PS mass loss; FTIR unchanged; Fig. S4). For exposures, Luria Bertani (LB)-grown cultures were washed and normalized in M9 to a wet biomass of ~0.03 g/ml (OD_600_/CFU equivalents in Text S3).

### Plate-based co-exposure (Mi-PS + Bacteria)

Bacterial suspensions (0.03 g/ml in M9 buffer) were mixed with serially diluted Mi-PS in M9 to yield final Mi-PS concentrations of 0, 1, or 10 mg/L. To minimize abiotic oxidation or prior biological modification of Mi-PS, mixtures were prepared immediately before plating. 2 × 2 cm^2^ lawn was spotted on 35-mm NGM plates (Fig. S5A). Synchronized worms were placed directly onto lawns for co-exposure and feeding (Fig. S5B). Exact timings and transfer schedules are in Text S4.

### Liquid co-incubations and volatile/oxygenate profiling

To evaluate microplastic–bacteria interactions, each strain was incubated in NGM containing 0.1% (w/v) Mi-PS for 48 h at 25°C with shaking (180 rpm). Resulting cultures were imaged by field-emission scanning electron microscopy (FE-SEM) and fluorescence microscopy (instrumentation in Text S5) and analyzed by solid-phase microextraction gas chromatography–mass spectrometry (SPME/GC–MS) for low-molecular-weight aromatic and oxygenated products (Fig. S6; instrument parameters, internal standardization, and spectral matching in Text S6). To determine whether metabolite production was specific to Mi-PS, we replaced Mi-PS with size-matched Mi-Si and analyzed cultures under identical conditions.

### Surface chemistry and molecular weight of Mi-PS

Mi-PS beads were recovered from bacterial incubations, cleared of adherent biomass using sequential acid, base, and detergent washes (HCl, NaOH, SDS), rinsed with alcohol, dried, and analyzed by FTIR and GPC. “Initial-time” controls were processed in parallel without bacterial incubation. Exact conditions and quality controls are summarized in Text S7.

### *C. elegans* lifespan assays

AU37 L4 worms (synchronized on OP50 to standardize developmental timing; Fig. S7) were transferred to lawns of OP50, LG3, or SCGB1 without microparticles and then moved to fresh plates seeded with the same strain and supplemented with Mi-PS or Mi-Si at 0, 1, or 10 mg/L (30 worms per plate; ≥3 plates per group). Survival was scored every 12 h, and plates were renewed every 48 h. Assay variants included exposures with live biomass, heat-killed biomass (Fig. S8), biofilm-induced biomass (Fig. S3), microbially pre-treated Mi-PS, and biomass supplemented with strain-specific metabolites. Detailed procedures are provided in Text S8.

### *C. elegans* oxidative stress analysis

Reactive oxygen species (ROS) were quantified after 50 h exposures (with/without 10 mg/L Mi-PS) using CM-H_2_DCFDA. Staining, anesthesia, imaging parameters (exposure, gain), and analytical settings are standardized across groups (details in Text S9).

### *C. elegans* growth rate and locomotion analysis

Body size (length/width), locomotion speed, and pharyngeal pumping were measured after 50 or 100 h of exposure (0/1/10 mg/L Mi-PS; 30 worms per plate; triplicates). Imaging and tracking (WormLab) used fixed thresholds and illumination corrections (Fig. S9). Pharyngeal pumping was counted visually over 1 min in randomly selected animals; a representative recording is included (Video S1). Analysis specifics are provided in Text S10.

### Quantification of bioaccumulated Mi-PS

Synchronized worms were exposed on strain-specific lawns (OP50, LG3, SCGB1) supplemented with Mi-PS (10 mg/L) for defined intervals (30 worms per plate; n = 3 plates). Animals were then transferred to Mi-PS–free lawns for 6 h, serially washed in fresh M9, rested for 10 h in M9 to purge unretained particles, and imaged under fixed settings (Fig. S10). Sixteen randomly selected worms per plate were quantified by fluorescence microscopy; the primary endpoint was luminal particle count per animal. Assay variants included live versus heat-killed bacterial biomass, DAF-pathway mutants (AA1, GR1307, AMH55; Fig. S11), LPS co-exposure (0–100 μg/ml; Fig. S12), and biomass supplemented with volatile metabolites. Detailed procedures are provided in Text S11.

### Quantification of DAF-12 and DAF-16 GFP nuclear localization

Nuclear localization of DAF-12::GFP (OH14589) and DAF-16::GFP (MQD1543) was quantified after acute exposure to volatile metabolites—isoamyl alcohol, isobutyrate, and isovalerate—at 0, 1, or 10 mM in NGM liquid medium (Fig. S13). Animals were synchronized to the L4 stage on OP50, washed in metabolite-containing NGM, exposed for 1 h, fixed with paraformaldehyde, and imaged on the same day under standardized acquisition settings. Nuclear puncta were scored as present/absent, and the proportion of animals exhibiting nuclear puncta was quantified (10–30 animals per group per experiment; three independent experiments). Detailed procedures are provided in Text S12.

### LPS release by gram-negative bacteria (LG3 vs OP50)

LG3 and OP50 were incubated with or without Mi-PS (10 mg/L) on NGM for 50 h at 25°C. Biomass was collected into 0.1% Triton X-100 solution, normalized by OD_600_, and assayed by a chromogenic LAL kit with calibration (Fig. S14). Conditions for extraction, and normalization appear in Text S13.

### Transcriptomics and quantitative real-time PCR (qPCR)

For RNA-seq, AU37 L1 worms (200 individuals per 90-mm plate) were exposed (0 or 10 mg/L Mi-PS; OP50, LG3, or SCGB1) for 100 h, washed to minimize bacterial carryover, and preserved in TRIzol. Library prep (rRNA depletion), sequencing (PE 2 × 100), preprocessing, alignment (WBcel235), quantification, and differential expression (edgeR, TMM) followed standard pipelines [42]. Fold-change filters (|FC| ≥ 2) were used for exploratory DE lists, with GO/KEGG enrichment (Benjamini–Hochberg) [42–44]. qPCR validation targeted *fmo-2* after Mi-PS or Mi-PS + LPS exposures; *snb-1* served as the reference. For LG3, *lpxA/C/D* expression was assessed after Mi-PS (0/1/10 mg/L) with *rpoA* as the reference. Primer sequences, thermocycling conditions, QC thresholds (RIN > 7; melt-curve checks), and platform details are provided in Text S14.

### Statistical analysis

Data are presented as mean ± SD unless otherwise stated; dots in plots denote individual replicates. For two-group comparisons, we used two-sided Welch’s t-test (α = 0.05). Where two-group tests were performed, *P* values were adjusted using the Holm–Šidák method. For multi-group comparisons, we used one-way ANOVA; when ANOVA was significant, Tukey’s HSD was used for all pairwise comparisons. Survival was analyzed using Kaplan–Meier curves and two-sided log-rank (Mantel–Cox) tests; where appropriate, Cox proportional hazards models were fitted to estimate hazard ratios (HR) with 95% CIs, and the proportional hazards assumption was checked. We report adjusted *P* values and consider results significant at adjusted *P* < .05. Significance in figures is annotated as *P* < .05 (^*^), *P <* .01 (^**^), *P <* 0.001 (^***^), *P <* .0001 (^****^); “ns” indicates not significant. Analyses were performed in GraphPad Prism 9.

## Results

### Bacterial strain modulates lifespan in *C. elegans* exposed to Mi-PS

C. elegans feeds on bacterial cells with diameters around 1 μm [45]. When 1-μm Mi-PS are co-exposed with bacterial cells, these particles are inadvertently ingested along with the diet and subsequently bioaccumulate (Video S2A–B). To validate host–bacteria–microplastic interactions, C. elegans were fed mixtures containing Mi-PS and phylogenetically distinct bacterial strains, including E. hormaechei LG3*,* B. amyloliquefaciens SCGB1, and E*.* coli OP50, each normalized to equivalent biomass levels [46]. LG3 and SCGB1 had previously been demonstrated to possess PS-degrading capacity under aerobic conditions [21], whereas OP50 lacked PS-degrading activity in our validation assay (Fig. S4). Mi-PS was titrated (0–10 mg/L) under the LG3 diet to define an exposure range for interaction analyses; lifespan was significantly reduced at concentrations qu mg/L, and 1 mg/L was therefore set as the minimum Mi-PS concentration for subsequent experiments (Fig. S15).

**Figure 1:**
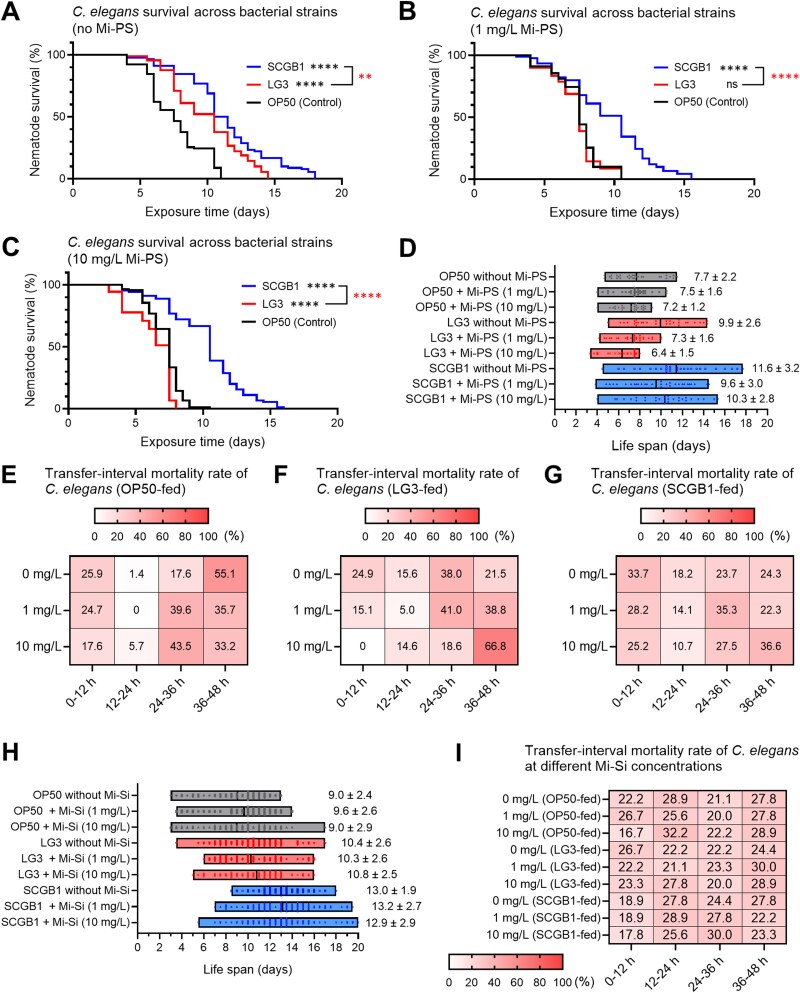
Bacterial strain-dependent effects on *C. elegans* survival under mi-PS exposure. (A–G) Worms were exposed to mi-PS by feeding on lawns prepared from mixtures of mi-PS and equalized bacterial biomass plated on NGM agar. Synchronized L4 *C. elegans* AU37 were fed *E. hormaechei* LG3, *B. amyloliquefaciens* SCGB1, or *E. coli* OP50 (control). (A–C) Kaplan–Meier survival curves at 0 mg/L mi-PS (a; without mi-PS), 1 mg/L (B), or 10 mg/L (C). (D) Mean lifespan (days ± SD) for all diet–dose combinations derived from the survival assays. (E–G) transfer-interval mortality profiles showing the percentage of deaths in each 12-h bin within a 48-h transfer cycle for worms fed OP50 (E), LG3 (F), or SCGB1 (G) at 0, 1, and 10 mg/L mi-PS. (H–I) non-polymeric particle control using mi-Si under the same conditions. (H) Mean lifespan (days ± SD) for all diet–dose combinations with mi-Si. (I) Transfer-interval mortality profiles for worms fed OP50, LG3, or SCGB1 at 0, 1, and 10 mg/L mi-Si.

Lifespan assays showed strain-specific effects contingent on Mi-PS (Fig. 1A–D). In the absence of Mi-PS, baseline lifespan ranked SCGB1 > LG3 > OP50. At 1 mg/L Mi-PS, mean lifespan decreased by 17.0% in SCGB1-fed worms (9.63 vs. 11.60 days) and by 26.2% in LG3-fed worms (7.32 vs. 9.92 days), whereas OP50-fed worms showed only a 2.7% decrease (7.18 vs. 7.68 days). At 10 mg/L Mi-PS, the reduction was greatest in the LG3-fed group (35.4%, 6.41 vs. 9.92 days), whereas SCGB1- and OP50-fed groups showed smaller decreases of 11.0% (10.33 vs. 11.60 days) and 6.5% (7.18 vs. 7.68 days), respectively. LG3-fed worms showed the most pronounced Mi-PS concentration–dependent reduction in lifespan among the tested diets, and Kaplan–Meier analysis confirmed this strain-specific dose relationship (Fig. S16A–C). In LG3-fed worms, survival decreased significantly with increasing Mi-PS concentration.

Interval-based mortality analysis revealed strain-specific temporal patterns (Fig. 1E–G). Under Mi-PS, LG3-fed worms exhibited a shift toward deaths in the 36–48 h interval that became more pronounced at higher Mi-PS concentrations, whereas SCGB1-fed and OP50-fed worms showed weaker and less concentration-dependent temporal shifts. When Mi-PS was replaced with size- and morphology-matched silica microspheres (Mi-Si), neither the concentration-dependent lifespan reduction nor the temporal mortality patterns were detected (Fig. 1H–I and Fig. S16D–F).

### Strain-specific physicochemical alterations and metabolite outputs of Mi-PS

**Figure 2:**
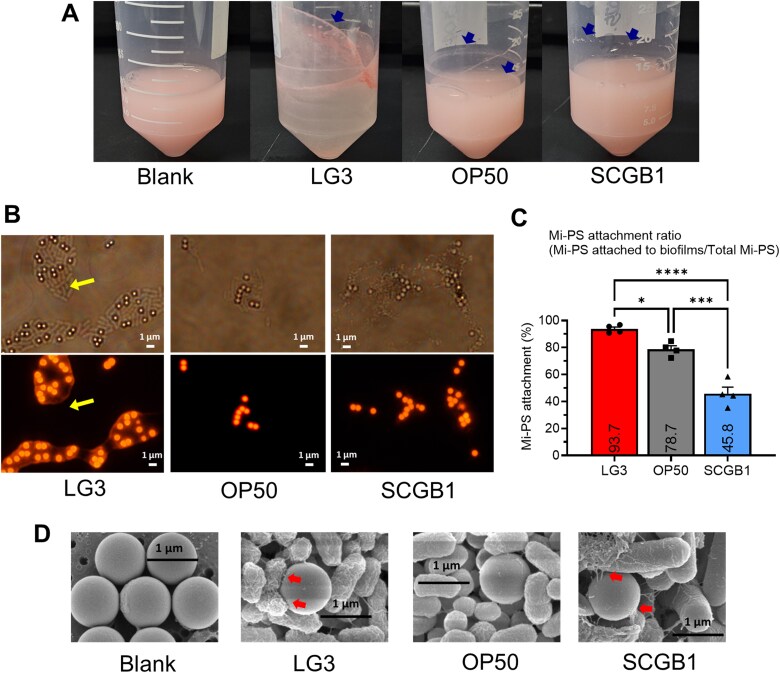
Strain-specific interactions with mi-PS under NGM conditions. (A) Representative images showing mi-PS aggregation and sedimentation after 48 h incubation in NGM. (B) Mi-PS attached to biofilms formed by strains LG3, OP50, and SCGB1. Top, bright-field images showing biofilm structures and mi-PS; bottom, fluorescence images showing the mi-PS signal. In the LG3 condition, mi-PS were entrapped within an amorphous extracellular matrix. Yellow arrows indicate biofilm regions where cells were present but the amorphous extracellular matrix was not apparent. (C) Attachment ratio of mi-PS to bacterial biofilms. (D) Representative FE-SEM images of mi-PS alone and after co-incubation with the bacterial strains LG3, OP50, and SCGB1.

To characterize bacteria–microplastic interactions in the absence of the host, Mi-PS was incubated with bacterial cell mass in NGM under oxic conditions, and particle-associated phenotypes, polymer chemistry, and metabolite outputs were analyzed. After incubation, visible aggregation and sedimentation of Mi-PS were observed in all bacterial treatments but not in the blank control, with the strongest aggregation in the LG3 group (Fig. 2A). Microscopy showed Mi-PS attached to bacterial biofilms; in LG3, an amorphous extracellular matrix encased and entrapped the particles (Fig. 2B). This matrix was localized around Mi-PS and was not observed in biofilms formed without Mi-PS (Fig. S17). Quantification of biofilm-attached Mi-PS showed clear strain dependence, with capture capacity ranked LG3 > OP50 > SCGB1 (Fig. 2C). In carbon-limited medium, Mi-PS supplementation promoted growth of both LG3 and SCGB1; however, the Mi-PS attachment ratio increased in SCGB1 but decreased in LG3 (Fig. S18). Despite these interaction phenotypes, FE-SEM revealed no overt physical degradation or structural alteration of Mi-PS after 48 h incubation with any strain, although pili-mediated attachment to Mi-PS was observed in the LG3 and SCGB1 groups (Fig. 2D).

**Figure 3:**
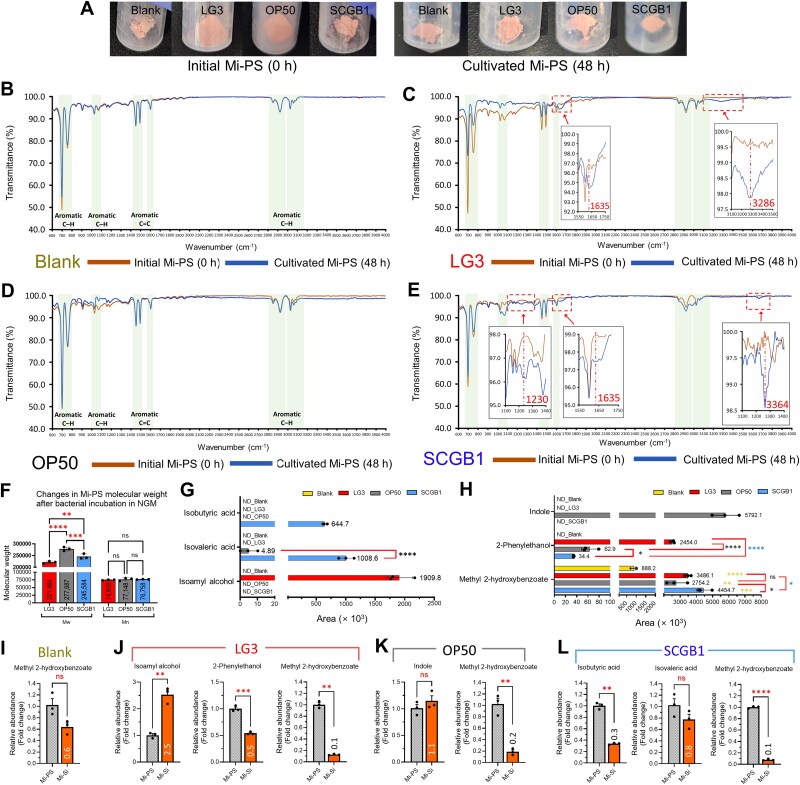
Strain-specific mi-PS oxidation and metabolite signatures. (A) Representative images of mi-PS pellets collected at 0 h and after 48 h co-incubation with blank, LG3, OP50, or SCGB1 following HCl/SDS-based removal of bacterial cells; the resulting cell-free pellets were used for downstream molecular profiling. (B–E) FTIR spectra of mi-PS before incubation (0 h) and after 48 h co-incubation with blank (B), LG3 (C), OP50 (D), or SCGB1 (E). (F) GPC-derived molecular weight of mi-PS after incubation across treatments. (G–H) SPME/GC–MS quantification of strain-associated metabolites after 48 h incubation in NGM containing 0.1% mi-PS: Aliphatic metabolites (G) and aromatic metabolites (H). (I–L) effect of replacing mi-PS with size- and morphology-matched mi-Si on strain-specific metabolite production. Relative abundance is shown as fold change, normalized to the corresponding mi-PS condition (mi-PS = 1), for blank (I), LG3 (J), OP50 (K), and SCGB1 (L).

After removing bacterial biomass, recovered particles were analyzed by FTIR and GPC to assess strain-specific chemical changes (Fig. 3A–F). In the blank control, Mi-PS spectra were unchanged before versus after incubation (Fig. 3B). By contrast, Mi-PS recovered from LG3 showed distinct spectral changes (Fig. 3C), including new absorption bands consistent with oxygen-containing functional groups (C=O around 1635 cm^−1^ and a broad O–H band in the 3100–3500 cm^−1^ region) [47–49], together with reduced intensities of PS benzyl ring–associated peaks (600–800, 1000–1100, and 1400–1500 cm^−1^) relative to the initial Mi-PS [21, 50–52]. Mi-PS recovered from OP50 showed only minor peak variations and no new bands indicative of functional group formation (Fig. 3D). In contrast, Mi-PS recovered from SCGB1 exhibited oxygen-containing features, including a C–O band around 1230 cm^−1^ and a broad O–H band in the 3600–3800 cm^−1^ region (Fig. 3E) [20, 49, 53]. GPC further revealed significant between-treatment differences in Mi-PS molecular weight, following the order OP50 > SCGB1 > LG3 (Fig. 3F). When these bacterial-pretreated Mi-PS particles were recovered and reintroduced into the *C. elegans* assay together with OP50 as the food source, Mi-PS pretreated with SCGB1 or LG3 induced a significant reduction in worm lifespan compared with OP50-pretreated Mi-PS (Fig. S19A and B). Among these groups, LG3-pretreated Mi-PS caused the greatest reduction in survival.

To profile strain-specific metabolites produced in the presence of Mi-PS, culture supernatants were analyzed by SPME/GC–MS (Fig. 3G and H). Isoamyl alcohol, isobutyric acid, isovaleric acid, 2-phenylethanol, and indole were detected only in bacterial treatments, whereas methyl 2-hydroxybenzoate was detected in both bacterial treatments and the blank but was higher in bacterial groups (SCGB1 > LG3 > OP50). Isovaleric acid was detected in OP50 and SCGB1 (∼200-fold higher in SCGB1 than OP50), isobutyric acid only in SCGB1, isoamyl alcohol only in LG3, indole only in OP50, and 2-phenylethanol in all bacterial treatments with 40–75-fold higher abundance in LG3 than in OP50 or SCGB1. Exposure to 10 mM 2-phenylethanol significantly shortened C. elegans lifespan, whereas 10 mM isoamyl alcohol had no significant effect (Fig. S19C and D).

When Mi-PS was replacedwith Mi-Si, metabolite outputs shifted in a strain-dependent manner (Fig. 3I–L): in LG3, isoamyl alcohol increased whereas 2-phenylethanol and methyl 2-hydroxybenzoate decreased; in OP50, indole did not change significantly whereas methyl 2-hydroxybenzoate decreased; and in SCGB1, isobutyric acid decreased, isovaleric acid did not change significantly, and methyl 2-hydroxybenzoate decreased (the blank showed no significant change). Overall, strain-dependent differences were observed in Mi-PS adhesion, polymer chemistry, and metabolite outputs, as well as in strain-specific metabolite shifts upon replacement with Mi-Si.

### Active bacterial interactions with Mi-PS drive strain-specific host oxidative stress and Mi-PS accumulation

**Figure 4:**
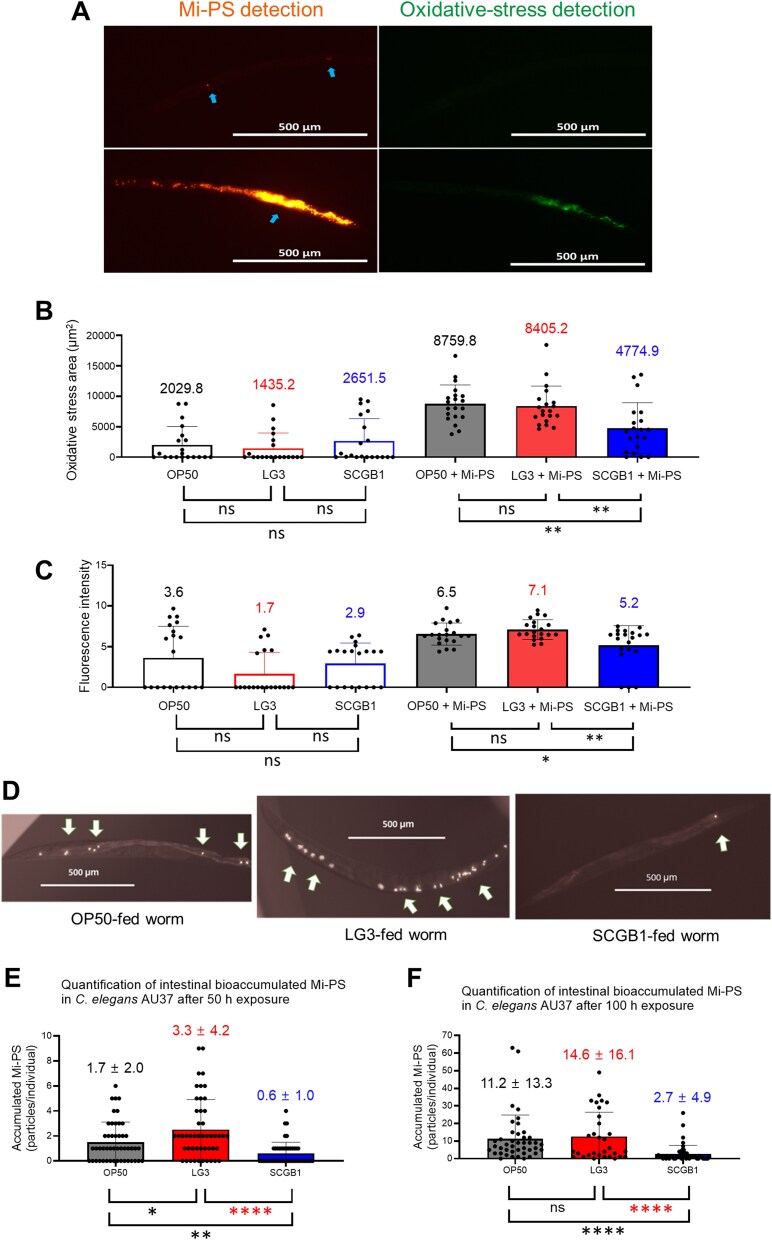
Strain-specific oxidative stress and mi-PS accumulation in *C. elegans* AU37. (A) Representative fluorescence microscopy images of worms exposed to mi-PS. (B–C) quantification of oxidative stress area and intensity in worms fed OP50, LG3, or SCGB1, with and without 10 mg/L mi-PS after 50 h of exposure. (D) Representative fluorescence microscopy images showing mi-PS particles (arrows) accumulated in the intestinal lumen of *C. elegans* after 100 h exposure to 10 mg/L mi-PS. (E–F) quantification of accumulated mi-PS particles in worms after 50 h or 100 h of exposure to live bacterial diets.

C. elegans ingested Mi-PS, and a subset of particles was retained in the intestinal lumen (Video S2). Mi-PS retention was accompanied by elevated oxidative stress, as measured by CM-H_2_DCFDA fluorescence (Fig. 4A). In the absence of Mi-PS, oxidative-stress signals did not differ among diets; upon Mi-PS exposure, oxidative-stress area and intensity increased across all diets, with the largest induction in LG3-fed worms and the smallest in SCGB1-fed worms (area ≈5.9-, 4.3-, and 1.8-fold; intensity ≈4.3-, 1.8-, and 2.1-fold for LG3, OP50, and SCGB1, respectively; Fig. 4B and C). Consistent with these trends, intestinal Mi-PS accumulation was diet dependent and increased over time, with retention highest in LG3-fed worms and lowest in SCGB1-fed worms at both 50 h and 100 h (Fig. 4D–F).

**Figure 5:**
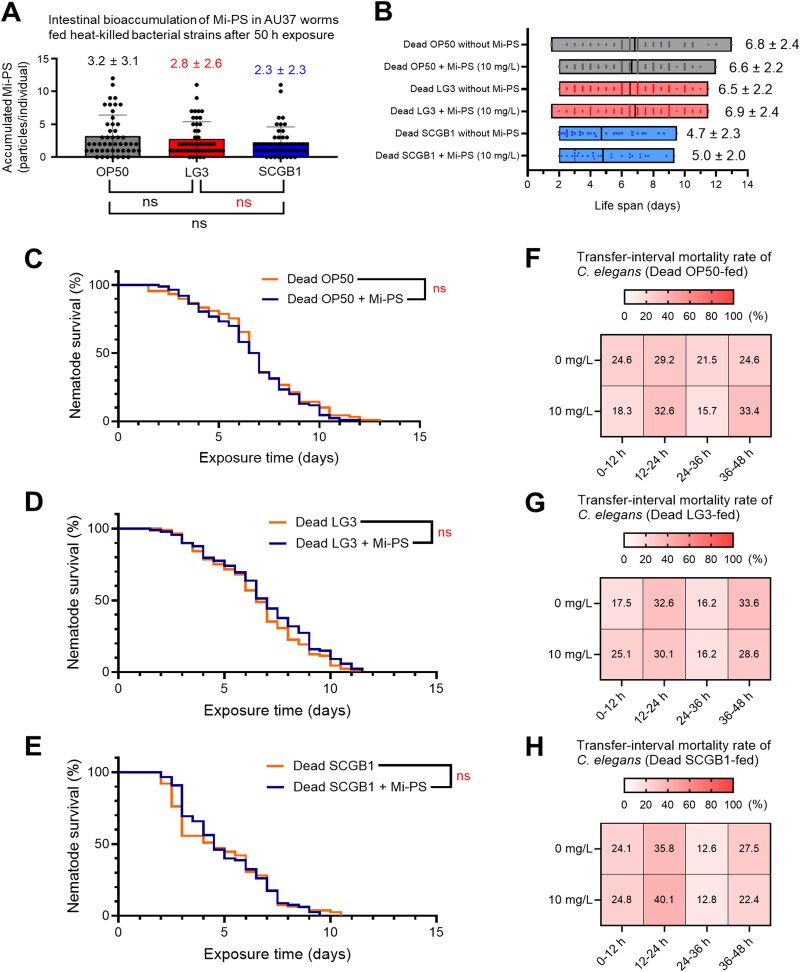
Heat-killed bacterial diets abolish strain-specific effects of mi-PS on toxicity. (A) Quantification of accumulated mi-PS in AU37 worms after 50 h of exposure to heat-killed bacterial diets. (B) Mean lifespan (days ± SD) of worms fed heat-killed bacterial cell mass with or without 10 mg/L mi-PS. (C–E) Kaplan–Meier survival curves of worms fed heat-killed OP50 (C), LG3 (D), or SCGB1 (E). (F–H) transfer-interval mortality profiles for worms.

Under heat-killed diets, strain-dependent differences in Mi-PS retention were lost (Fig. 5A), and the lifespan-shortening effects of Mi-PS observed with live bacteria were abolished (Fig. 5B–E). Likewise, the strain-specific temporal mortality patterns across transfer intervals disappeared under heat-killed diets (Fig. 5F–H). Together, these data show that the strain-dependent patterns in Mi-PS retention and survival require live bacterial activity.

### Differential physiological impacts of Mi-PS exposure in *C. elegans* fed with distinct bacterial strains

**Figure 6:**
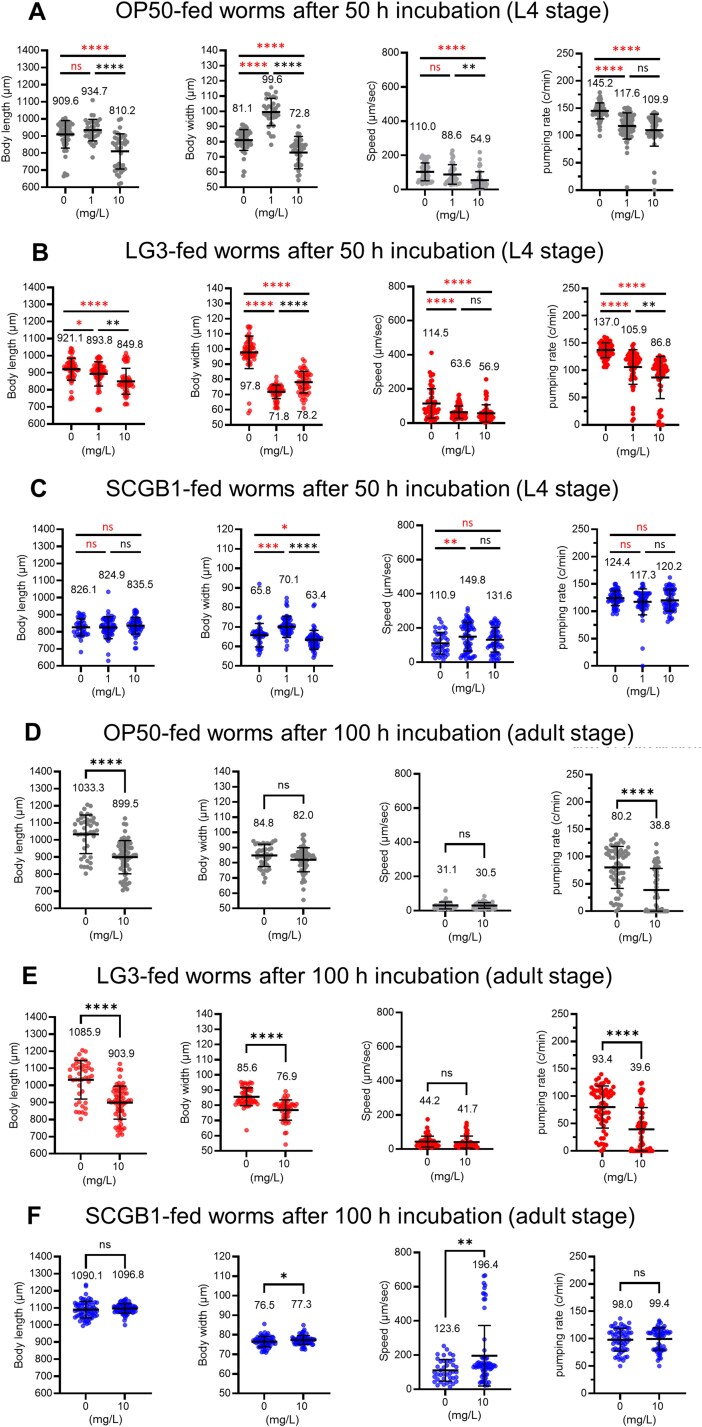
Strain-dependent physiological impairments in *C. elegans* after 50 h or 100 h of mi-PS exposure. Body length, body width, locomotion speed, and pharyngeal pumping were measured in worms fed with LG3 (a, D), OP50 (B, E), or SCGB1 (C, F) at 0, 1, or 10 mg/L mi-PS for 50 h (A–C) or 100 h (D–F).

To assess how strain-specific Mi-PS interactions shape host physiology, L1 larvae (~250 μm) were exposed to equalized bacterial biomass supplemented with Mi-PS. Comparative analyses revealed that Mi-PS exposure impaired worm growth and behavior to varying degrees depending on the bacterial strain (Video S3). These physiological impairments were particularly evident in the Gram-negative strains E. hormaechei LG3 and E. coli OP50 (Fig. 6A and B). After 50 h of exposure, both LG3- and OP50-fed worms exhibited reductions in body length, body width, locomotion, and pharyngeal pumping under Mi-PS exposure, though the effects were more consistent and dose dependent in LG3-fed worms. By contrast, in SCGB1-fed worms, physiological effects of Mi-PS exposure were largely negligible (Fig. 6C); body length, locomotion, and pharyngeal pumping showed no significant decreases with increasing Mi-PS concentration.

After 100 h of continuous exposure, deficits persisted and were more pronounced in LG3- and OP50-fed worms (Fig. 6D and E). At this adult-stage time point (body length > 900 μm), 43.5% of LG3-fed worms and 42.9% of OP50-fed worms remained <900 μm under Mi-PS exposure. Individuals shorter than 800 μm exhibited reduced locomotor activity (Videos S3B, F) and pronounced intestinal Mi-PS accumulation (Fig. S20). By contrast, SCGB1-fed worms showed no significant impairments in any parameter (Fig. 6F). In physiological assessment, continuous Mi-PS exposure from immediately after hatching in C. elegans adversely affected feeding capacity, growth rate, and locomotion. The magnitude of impairment varied with the co-supplied bacterial strain.

### Transcriptomic reprogramming and *daf* signaling govern strain-specific responses to Mi-PS

**Figure 7:**
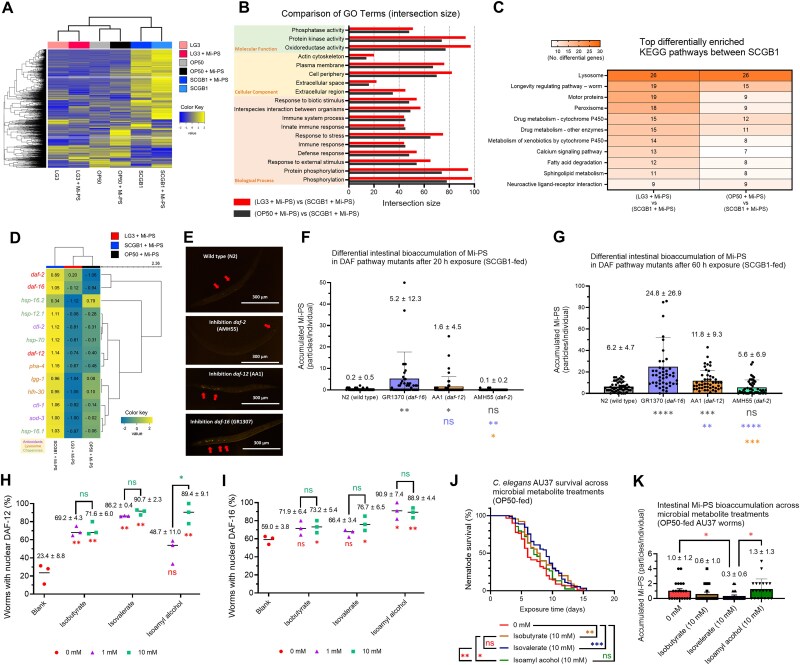
Strain-dependent transcriptomic programs and *DAF* signaling govern mi-PS responses in *C. elegans*. (A) Hierarchical clustering heatmap of DEGs in AU37 worms fed OP50, LG3, or SCGB1, with or without 10 mg/L mi-PS exposure. (B) GO enrichment of DEGs comparing SCGB1-fed worms with LG3-fed or OP50-fed worms under mi-PS exposure, highlighting top terms across biological process, cellular component, and molecular function categories. (C) KEGG pathway enrichment major showing pathways differentially enriched in SCGB1-fed worms relative to LG3- or OP50-fed worms under mi-PS exposure. (D) Heatmap of *DAF* signaling components (*DAF-2*, *DAF-12*, *DAF-16*) and downstream targets associated with autophagy–lysosome function, antioxidant defense, and molecular chaperones. (E) Representative fluorescence micrographs of wild-type N2 and *DAF* mutants (GR1307, *DAF-16*; AA1, *DAF-12*; AMH55, *DAF-2*) fed SCGB1 and exposed to 10 mg/L mi-PS; luminally retained mi-PS particles are indicated by arrows. (F, G) quantification of intestinal mi-PS accumulation after 20 h (F) or 60 h (G) exposure in N2 and *DAF* mutants under the SCGB1 diet. (H, I) fraction of animals showing nuclear localization of DAF-12::GFP (OH14589; H) and DAF-16::GFP (MQD1543; I) after exposure to strain-specific metabolites. (J) Kaplan–Meier survival curves of AU37 worms under mi-PS exposure in the presence of metabolites that induce DAF-12/DAF-16 nuclear localization. (K) Quantification of intestinal mi-PS accumulation in AU37 after 50 h exposure across metabolite treatments.

To probe mechanisms underlying divergent physiological outcomes, we conducted RNA-seq on worms exposed to biomass-normalized bacterial diets with or without Mi-PS (10 mg/L). Hierarchical clustering showed that global expression patterns clustered by bacterial strain rather than by Mi-PS treatment (Fig. 7A), indicating that diet strain is the major determinant of the transcriptional landscape. Under the shared condition of Mi-PS exposure, we then conducted gene ontology (GO) enrichment using differentially expressed genes (DEGs) from pairwise comparisons of SCGB1-fed worms versus LG3-fed or OP50-fed worms (Figs S21–S23).

Overall, enriched GO terms were broadly similar between the LG3–SCGB1 and OP50–SCGB1 comparisons, whereas enrichment magnitudes were generally greater in the LG3–SCGB1 comparison (Fig. 7B). In the Biological Process category, phosphorylation and protein phosphorylation were enriched, together with terms related to response to external stimulus, defense response, and immune response. In the Cellular Component category, DEGs were enriched in the cell periphery, plasma membrane, and actin cytoskeleton [54, 55]. In the Molecular Function category, enriched terms were largely associated with catalytic and signaling activities, including oxidoreductase activity, protein kinase activity, and phosphotransferase activity. Together, these results show that under Mi-PS exposure, SCGB1-fed worms exhibit a transcriptional program distinct from LG3- and OP50-fed worms, including enrichment of phosphorylation-related signaling terms and redox/oxidoreductase-associated functions.

KEGG enrichment analysis identified pathway-level differences under Mi-PS exposure in lysosome, longevity, peroxisome, metabolism of xenobiotics by cytochrome P450, and calcium signaling pathways, with LG3- and OP50-fed worms differing from SCGB1-fed worms (Fig. 7C; Fig. S24). The contrast was more pronounced in the LG3–SCGB1 comparison. Within the longevity regulating pathway, *daf-2*, *daf-16*, and *daf-12*, along with downstream targets associated with the autophagy–lysosome system (*pha-4*, *lgg-1*, *hlh-30*), antioxidant defense (*ctl-1*, *ctl-2*, *sod-3*), and molecular chaperones (*hsp-16.1*, *hsp-16.2*, *hsp-12.1*, *hsp-70*), were consistently elevated in SCGB1-fed worms compared with LG3- or OP50-fed worms (Fig. 7D). However, in SCGB1-fed worms, expression levels of these genes were similar regardless of Mi-PS treatment (fold change <2; Table S2).

We quantified intestinal Mi-PS bioaccumulation across *daf* mutants under the SCGB1 diet to test the role of *daf* signaling in the Mi-PS response (Fig. 7E). After 20 h, GR1307 showed the highest accumulation, followed by AA1, N2, and AMH55 (Fig. 7F). After 60 h, GR1307 and AA1 retained significantly more Mi-PS than N2, with GR1307 showing the greatest accumulation; AMH55 showed slightly lower accumulation than N2, although this difference was not significant (Fig. 7G).

Using strain-specific metabolites identified by SPME–GC/MS, we evaluated pathway engagement in DAF-12::GFP and DAF-16::GFP reporters. Isobutyrate, isovalerate, and isoamyl alcohol increased the fraction of animals showing nuclear localization of both DAF-12 and DAF-16 at 10 mM (Fig. 7H and I). Under identical conditions with Mi-PS exposure (10 mg/L), isobutyrate and isovalerate significantly improved AU37 survival, whereas isoamyl alcohol showed limited benefit (Fig. 7J). Consistent with these outcomes, isovalerate significantly reduced intestinal Mi-PS accumulation relative to the other treatments (Fig. 7K).

### Mi-PS exposure stimulates bacterial LPS biosynthesis and promotes host xenobiotic/oxidative stress

**Figure 8:**
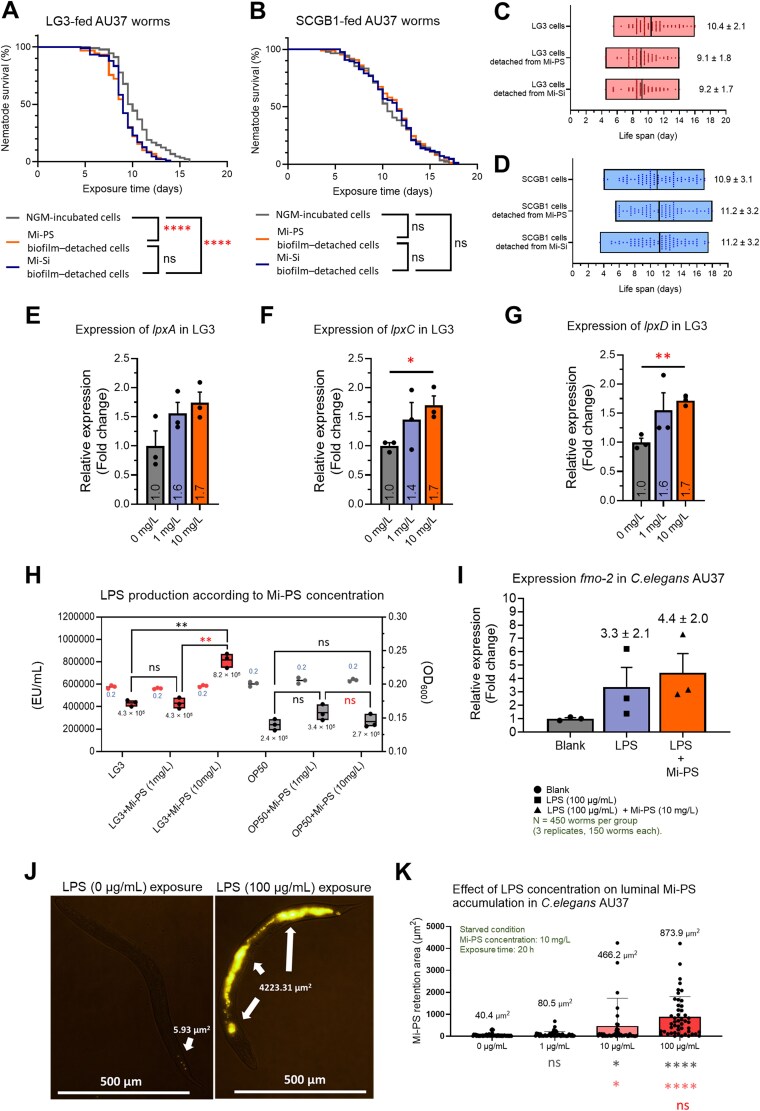
Mi-PS promotes particle-associated biofilm formation and LPS biosynthesis in LG3, leading to elevated host stress signaling and increased intestinal mi-PS retention. (A–D) survival analysis of AU37 worms fed biofilm-derived cells. Particle-associated biofilms were induced using 25 μm mi-PS or mi-Si, after which biofilm-derived bacterial cells were isolated and fed to AU37 worms. Survival was compared between worms fed LG3-derived or SCGB1-derived cells. (E–G) relative expression of LPS biosynthesis genes (*lpxA*, *lpxC*, *lpxD*) in LG3 cultured with 0, 1, or 10 mg/L mi-PS for 48 h. (H) Quantification of LPS production in LG3 and OP50 cultured with increasing concentrations of mi-PS. (I) Expression of the xenobiotic/oxidative stress reporter *fmo-2* in AU37 worms exposed to purified LPS (100 μg/ml), with or without 10 mg/L mi-PS. (J) Representative fluorescence images showing luminal mi-PS accumulation in AU37 worms exposed to 0 or 100 μg/ml purified LPS in the absence of bacterial biomass; arrows indicate retained mi-PS particles. (K) Quantification of intestinal mi-PS accumulation in AU37 worms exposed to mi-PS together with increasing concentrations of purified LPS.

Compared with SCGB1-fed worms, LG3- and OP50-fed worms showed higher induction of the xenobiotic/oxidative stress reporters *fmo-2* and *gst-4* in response to Mi-PS (Fig. S25) [56–58]. Our previous work showed that LG3 can utilize Mi-PS as a carbon source under carbon-limited conditions and concomitantly upregulated LPS biosynthetic genes (*lpxA*, *lpxC*, *lpxD*) [21]. After inducing particle-associated biofilms with Mi-PS or Mi-Si and isolating biofilm-derived cell mass, LG3 biofilm-detached cells reduced AU37 survival regardless of particle type, whereas SCGB1 biofilm-detached cells had no detectable effect (Fig. 8A–D).

Because LG3 formed particle-associated biofilms and exhibited distinct Mi-PS entrapment patterns in NGM, we examined LPS-related responses under these conditions. LG3 incubated with Mi-PS showed increased expression of LPS biosynthesis genes (Fig. 8E–G), with significant upregulation of *lpxC* and *lpxD* at 10 mg/L Mi-PS. Consistent with these transcriptional changes, LPS abundance was significantly higher in LG3 at 10 mg/L Mi-PS than at 0 mg/L (Fig. 8H), whereas OP50 showed no significant change in LPS production. To directly assess LPS effects on the host in the absence of bacterial biomass, worms were exposed to purified LPS. Exposure to 100 μg/ml LPS tended to increase *fmo-2* expression by 3.30 ± 2.07-fold compared with the untreated control (*P* = .245), and co-exposure to 10 mg/L Mi-PS plus 100 μg/ml LPS yielded a 4.38 ± 2.03-fold increase (*P =* .139), although neither comparison reached significance (Fig. 8I). Intestinal Mi-PS accumulation increased in a concentration-dependent manner with elevated LPS levels (Fig. 8J and K). In the absence of bacterial biomass, worms exhibited greater Mi-PS ingestion and intestinal retention than under bacterial co-feeding, and retention further increased with higher LPS concentrations.

## Discussion

In this study, Mi-PS toxicity in C. elegans AU37 was strongly dependent on the co-supplied bacterial strain, with the most pronounced lifespan and temporal mortality shifts observed under the LG3 diet. Strain-specific interactions with Mi-PS were reflected in differential particle adhesion/biofilm association, polymer chemical signatures, and metabolite outputs, which aligned with diet-dependent differences in intestinal Mi-PS retention, oxidative stress, and host physiology. Transcriptomic and functional assays further implicated *daf* signaling and LPS-associated responses as key modulators of these strain-specific outcomes.

To date, numerous bacteria capable of plastic biodegradation have been reported, many of which were isolated from the gut of organisms such as mealworms, waxworms, and superworms [20, 29, 59]. However, the effects of ingested microplastics on the metabolism and physiology of plastic-degrading bacteria, as well as the extent to which microbial biodegradation alters particle toxicity to the host, remain largely unknown [60–62]. To address this gap, we compared two previously validated PS-degrading strains, E*.* hormaechei LG3 and B*.* amyloliquefaciens SCGB1 [21], with a non-degrading strain, E*.* coli OP50, under Mi-PS exposure in C. elegans.

Mi-PS exposure reduced C. elegans lifespan in a strain-dependent manner, with the strongest effect observed in LG3-fed worms, followed by SCGB1-fed and OP50-fed worms. LG3-fed worms displayed a clear concentration-dependent decline, with mortality peaking at 36–48 h under the 48-h transfer regime, whereas heat-killed LG3 eliminated this pattern. These results indicate that Mi-PS exposure in the presence of live LG3, which involves both particle-level physicochemical changes and bacterial cellular responses, directly contributes to host mortality [63, 64]. Consistent with this interpretation, LG3 displayed several distinctive responses after 48 h incubation with Mi-PS. LG3 formed robust particle-associated biofilms, featuring pili-mediated attachment and an amorphous extracellular matrix that enveloped and physically entrapped Mi-PS, yielding the highest particle attachment among the tested strains. Moreover, biofilm-detached LG3 cell mass shortened AU37 lifespan relative to planktonic LG3, indicating that the particle-associated state of LG3 can enhance host toxicity beyond baseline diet effects. In parallel, Mi-PS interaction induced LPS-associated responses, including increased expression of LPS biosynthesis genes and elevated LPS abundance, which contribute to enhanced intestinal particle retention and may affect downstream host stress responses [65]. However, the biofilm-detached LG3 cell mass shortened host lifespan to a similar extent regardless of whether the biofilm was formed with Mi-PS (12.4% reduction) or Mi-Si (11.3% reduction) relative to planktonic LG3. This indicates that the lifespan decrease attributable to the biofilm-associated state is more consistent with a microparticle-general effect rather than a microplastic-specific effect. In contrast, co-exposure to live LG3 with Mi-PS at 10 mg/L reduced host lifespan by 26.2% compared with the no–particle condition, suggesting the presence of additional microplastic–specific toxicity determinants beyond biofilm formation alone.

LG3 formed particle-associated biofilms that effectively entrapped Mi-PS and was associated with pronounced changes in Mi-PS molecular chemistry as well as the production of specific metabolites. Although no clear structural alteration of Mi-PS was detected after the relatively short 48-h incubation, we observed discernible chemical modifications. In particular, FTIR analysis revealed the emergence of oxygen-containing functional groups on the Mi-PS surface, including C=O and O–H features characteristic of oxidative modification [47–49]. These spectral signatures closely resembled those reported for photo-oxidized polystyrene, suggesting that LG3-mediated oxidation increases polymer chemical reactivity and may compromise polymer integrity [47]. Oxidized particles have been reported to interact more strongly with biological membranes and to exhibit higher toxicity [66, 67]. Consistent with this, LG3-pretreated Mi-PS elicited a more pronounced lifespan reduction than Mi-PS pretreated with the other tested strains.

Metabolite profiling further supports the possibility that LG3 influences host outcomes through secreted small molecules. Under Mi-PS exposure in NGM, LG3 produced a distinct metabolite signature that included isoamyl alcohol, 2-phenylethanol, and methyl 2-hydroxybenzoate. Isoamyl alcohol was sufficient to induce nuclear localization of DAF-12 and DAF-16, but it did not significantly alter AU37 survival under Mi-PS exposure. Isoamyl alcohol has been reported to promote longevity in C. elegans via *daf* signaling [68, 69], which may reflect differences in genetic background and Mi-PS co-exposure. In contrast, in our assays, 2-phenylethanol significantly reduced C. elegans lifespan. 2-phenylethanol and methyl 2-hydroxybenzoate have been reported as styrene-derived metabolites in bacterial styrene metabolism [50, 70], and their abundances increased under Mi-PS relative to Mi-Si in our system. Given reports that oxidized styrene intermediates can contribute to toxicity [13, 71, 72], these metabolites warrant consideration as potential drivers of host stress under Mi-PS exposure.

Collectively, under Mi-PS exposure, LG3 presents three major factors that could increase host oxidative stress and reduce survival: (i) biofilm-mediated particle entrapment coupled with induction of LPS-associated responses, (ii) increased production of styrene-associated metabolites, and (iii) bio-oxidative modification of Mi-PS. Thus, LG3-specific processes induced under Mi-PS exposure could intensify host oxidative stress, compromise intestinal barrier integrity (Video S1B), and ultimately increase luminal retention of Mi-PS relative to other bacterial strains [37].

SCGB1-fed worms showed attenuated toxicity induced by Mi-PS. Although B. amyloliquefaciens SCGB1 can oxidize Mi-PS, host outcomes differ from those under the LG3 diet. Consistent with this phenotype, CM-H_2_DCFDA staining showed that Mi-PS increased oxidative-stress signals far less in SCGB1-fed worms than in LG3-fed worms (1.8-fold vs 5.9-fold). Transcriptomic profiling provides a plausible basis for this protection. RNA-seq analyses indicated that SCGB1-fed worms engaged transcriptional programs distinct from LG3- and OP50-fed worms, with enriched GO categories spanning phosphorylation-related signaling and redox-associated terms. KEGG analysis further supported this distinction, revealing divergence in lysosomal and longevity regulating pathways across strains. SCGB1-fed worms consistently exhibited higher expression of *daf-2*, *daf-16*, and *daf-12*, together with downstream genes linked to the autophagy–lysosome system, antioxidant defense, and molecular chaperones, relative to OP50- and LG3-fed worms [73].

The DAF-2/DAF-16 axis plays a central role in regulating oxidative-stress resistance and longevity in C. elegans [74, 75], and prior studies have also implicated DAF-2 and DAF-16 in modulating sensitivity to microplastic toxicity [18, 76]. Using *daf* mutant lines, we further confirmed that *daf-12* and *daf-16* modulate intestinal Mi-PS accumulation. Moreover, among strain-specific metabolites, isovalerate and isobutyrate altered DAF-12 and DAF-16 nuclear localization and improved survival under Mi-PS exposure; isovalerate also reduced intestinal particle accumulation, highlighting it as a candidate small molecule with potential relevance for mitigating Mi-PS toxicity. Metabolite profiling showed higher isobutyrate under Mi-PS than under Mi-Si (*P <* .05), with a non-significant increase in isovalerate (*P =* .161). This pattern supports the idea that Mi-PS exposure can enhance protective metabolite production and may contribute to attenuated toxicity in SCGB1-fed worms.

Nutrient context altered the interactions between PS-degrading bacteria and Mi-PS particles. In nutrient-rich NGM, LG3 exhibited a higher Mi-PS attachment ratio than SCGB1. Mi-PS particles in NGM can provide a structured microenvironment that promotes microcolony formation and the accumulation of extracellular polymeric substances, thereby locally concentrating water and nutrients and creating a favorable niche [77, 78]. By contrast, under carbon-limited conditions, SCGB1 showed a pronounced increase in biofilm formation and Mi-PS attachment, reaching levels comparable to LG3 (Fig. S18D). Under this condition, Mi-PS may additionally gain value as a potential carbon source, and effective degradation and utilization are expected to involve pili/fimbriae-mediated adhesion and biofilm formation [79]. Together, these results suggest that the ecological value of Mi-PS is context dependent, shifting the balance between “surface niche” and “resource” functions across nutrient conditions.

OP50, although non-degrading, still interacted physically with Mi-PS, forming mixed biofilms without evidence of physicochemical modification of Mi-PS. In OP50 cultures grown in NGM, indole production remained comparable regardless of particle type (Mi-PS vs Mi-Si). In contrast, methyl 2-hydroxybenzoate increased in the presence of Mi-PS (relative to Mi-Si; 5.26-fold change). These observations indicate that even bacteria lacking PS-degrading capacity can interact with Mi-PS and undergo metabolic and physiological shifts in response to particle exposure. Prior studies show that PS particles can alter E. coli membrane properties and stress responses [80, 81]. Consistent with this, Mi-PS exposure induced a significant lifespan reduction in OP50-fed worms only when live OP50 cells were provided, and was accompanied by increased expression of *fmo-2* and *gst-4*, two oxidative-stress markers [56–58]. Further studies are needed to elucidate how non-degrading bacteria physiologically adapt to microplastic exposure and the feedback of these adaptations on host health.

In conclusion, our findings demonstrate that host outcomes following microplastic exposure vary depending on the interacting bacterial strain, and that strain-derived metabolites capable of modulating host signaling, such as isobutyrate and isovalerate, contribute to protection by regulating DAF-12 and DAF-16. Beyond particle-intrinsic effects, our data show that bacterial physiological and metabolic responses to Mi-PS are major determinants of host stress. Under Mi-PS exposure, LG3 generated bio-oxidatively modified Mi-PS and produced styrene-associated oxidative metabolites, including 2-phenylethanol, which together align with reduced host survival. In parallel, Mi-PS interaction promoted a particle-associated lifestyle in LG3 and induced LPS-associated responses, including upregulation of core LPS biosynthesis genes (*lpxA*, *lpxC*, and *lpxD*), consistent with increased LPS output. Purified LPS exposure recapitulated key host responses to Mi-PS in C. elegans AU37, including *fmo-2* induction and enhanced intestinal Mi-PS accumulation. These data support a model in which bacteria act as intermediaries that modulate microplastic toxicity through metabolite-driven host signaling and LPS-linked stress pathways, rather than microplastics acting solely as inert particles. Microplastic toxicity is therefore shaped by dynamic interactions among the host, commensal bacteria, and particles. These insights have important implications for refining mechanistic risk-assessment frameworks and for developing microbiome-based strategies to mitigate the biological impacts of microplastic pollution.

## Supplementary Material

Supplemenarty_Materials_260307_final_wrag051

Video_S1_revised_wrag051

Video_S2_wrag051

Video_S3_wrag051

## Data Availability

The datasets supporting the conclusions of this article are publicly available. RNA sequencing data have been deposited in the NCBI Sequence Read Archive under accession numbers SRR33313652 and SRR33313653. The whole-genome sequencing data are available under BioProject accession number PRJNA1255511 and BioSample accession number SAMN48146405.
