# Supplementary material for: Polystyrene-degrading bacteria modulate host stress and toxicity responses to microplastic exposure in Caenorhabditis elegans
Source: ISME J. 2026 Mar 10;20(1):wrag051. doi: 10.1093/ismejo/wrag051 (PMC13064669; doi:10.1093/ismejo/wrag051)
Supplement: Supplemenarty_Materials_260307_final_wrag051 [file supplemenarty_materials_260307_final_wrag051.pdf]

Supplementary Materials

Polystyrene-degrading bacteria modulate host stress and toxicity responses to microplastic exposure in *Caenorhabditis elegans*

Min-Geun Kang, Daniel Junpyo Lee, Arthur Junghun Kim, Ki Beom Jang, Anna Kang, Youbin Choi, Jihyun Yoon, Eunsol Seo, and Younghoon Kim*

Department of Agricultural Biotechnology and Research Institute of Agriculture and Life Science, Seoul National University, Seoul 08826, Republic of Korea

*To whom correspondence should be addressed: ykeys2584@snu.ac.kr (+82-2-880-4808)

This supporting Information provides:

Total number of tables:2

Total number of texts:14

Total number of figures:25

Total number of pages:47

Supplementary video legends:3
Supplementary references:36

Correspondence: ykeys2584@snu.ac.kr; Tel: +82-2-880-4808

Contents

Supplementary Tables

Table S1. Key reagents and resources....S4

Table S2. DAF signaling and downstream gene expression in SCGB1-fed worms....S6

Supplementary Texts

Text S1. Preparation of biofilm-induced bacterial biomass using 25 μm microparticles....S7

Text S2. M9 buffer and Modified Nematode Growth Medium (NGM)....S7

Text S3. Standardization of bacterial cell mass....S8

Text S4. Plate-based co-exposure (Mi-PS + Bacteria)....S8

Text S5. Field-emission scanning electron microscopy (FE-SEM) and Fluorescence Microscopy....S8

Text S6. Solid-phase microextraction gas chromatography–mass spectrometry (SPME/GC–MS)....S9

Text S7. Surface chemistry and molecular weight of Mi-PS....S10

Text S8. *C. elegans* lifespan assays....S10

Text S9. C. elegans oxidative stress analysis....S12

Text S10. Growth rate and locomotion analysis....S13

Text S11. Quantification of luminally accumulated Mi-PS in *C. elegans*....S13

Text S12. Quantification of DAF-12 and DAF-16 GFP nuclear localization....S16

Text S13. Assessment of endotoxin release during Mi-PS exposure....S16

Text S14. Transcriptomics and qPCR S16

Supplementary Figures

Fig. S1. Morphological and physicochemical characterization of 1-μm Mi-PS....S19

Fig. S2. Size-matched silica microspheres (Mi-Si) used as an inert control for Mi-PS....S20

Fig. S3. Isolation of microparticle-induced biofilm–detached cells....S21

Fig. S4. Non-degradative property of *E. coli* OP50 toward PS....S22

Fig. S5. Experimental setup for co-exposure of *C. elegans* to bacteria and Mi-PS....S23

Fig. S6. Representative SPME/GC–MS chromatograms and annotated metabolites....S25

Fig. S7. Adult onset and egg-laying period under feeding conditions........S25

Fig. S8. Preparation of heat-killed bacterial cell mass........S26

Fig. S9. Workflow and imaging setup for growth and locomotion assays........S27

Fig. S10. Workflow for quantification of bioaccumulated Mi-PS in the gut........S28

Fig. S11. Representative phenotype of ault-stage N2 and daf mutant........S29

Fig. S12. Quantification of luminal Mi-PS under LPS exposure S30

Fig. S13. Detection of DAF reporter nuclear localization after metabolite exposure........S31

Fig. S14. Standard curve used for endotoxin quantification by the chromogenic LAL assay........S32

Fig. S15. Lifespan of *C. elegans* exposed to graded Mi-PS doses under LG3 feeding........S33

Fig. S16. Lifespan of *C. elegans* exposed to Mi-PS or Mi-Si under different bacterial diets........S34

Fig. S17. The amorphous extracellular matrix developed around the Mi-PS........S35

Fig. S18. Bacterial growth and Mi-PS aggregation characteristics........S36

Fig. S19. Strain-specific effects of pretreated Mi-PS and volatile metabolites on lifespan........S37

Fig. S20. Morphological variation and luminal Mi-PS accumulation........S38

Fig. S21. Enriched GO terms – Biological Process S39

Fig. S22. Enriched GO terms – Cellular Component S40

Fig. S23. Enriched GO terms – Molecular Function S41

Fig. S24. KEGG pathway enrichment analysis under Mi-PS exposure........S42

Fig. S25. Expression of oxidative stress reporters (*fmo-2*, *gst-4*)........S43

Supplementary video legends........S44

Supplementary references........S45

Supplementary Tables

Table S1. Key reagents and resources

Reagent or Resource	Source	Identifier
**Experimental models: Organisms/strains**		
C. elegans strain AU37 *glp-4(*bn2) I; *sek-1(*km4) X	Caenorhabditis Genetics Center (CGC)	WB-STRAIN: WBStrain00000261
C. elegans strain N2 (Bristol, wild type)	CGC	WB-STRAIN: WBStrain00000001
C. elegans strain AA1 *daf-12*(rh257) X	CGC	WB-STRAIN: WBStrain00000005
C. elegans strain GR1307 [*daf-16*(mgDf50) I	CGC	WB-STRAIN: WBStrain00007895
C. elegans strain AMH55 *daf-2*(e1370) III; *otIs117* IV	CGC	WB-STRAIN: WBStrain00047192
C. elegans strain OH14589 daf-12(ot870[daf-12::GFP::3xFlag])	CGC	WB-STRAIN: WBStrain00031132
C. elegans strain MQD1543 daf-16(hq23[daf-16::GFP])	CGC	WB-STRAIN: WBStrain00051774
**Bacterial strains**		
E. coli OP50	CGC	WB-STRAIN: WBStrain00041971
B. amyloliquefaciens SCGB1	Microbial Institute for Fermentation Industry (Sunchang, Korea)	N/A
E. hormaechei LG3	This study (isolated from *Tenebrio molitor* gut)	N/A
**Chemicals, peptides, and recombinant proteins**		
1 µm polystyrene microspheres (Mi-PS)	Polysciences, Inc.	Cat# 18660-5
1 µm silica microspheres (Mi-Si)	Polysciences, Inc.	Cat# 24326
25 µm polystyrene microspheres	Polysciences, Inc.	Cat# 18241
25 µm silica microspheres	GETNANO	Cat# SIO2-123-50G
CM-H_2DCFDA	Invitrogen (Thermo Fisher)	Cat# C6827
Sodium azide	Sigma-Aldrich	Cat# S2002; CAS: 26628-22-8

Lipopolysaccharides from *E. coli* O111:B4	Sigma-Aldrich	Cat# L4391
5-Fluoro-2′-deoxyuridine (FUdR)	Sigma-Aldrich	Cat# F0503; CAS: 50-91-9
**Critical commercial assays**		
RNeasy Mini Kit (RNA extraction)	Qiagen	Cat# 74104
iScript cDNA Synthesis Kit (cDNA synthesis)	Bio-Rad	Cat# 1708891
RealHelix™ Premier qPCR Kit (SYBR Green, Low ROX)	NanoHelix	Cat# PQL-S500
Qubit RNA HS Assay Kit	Invitrogen	Cat# Q32855
**Deposited data**		
RNA-seq raw reads from *C.elegans* AU37	NCBI SRA	SRR33313653 SRR33313652
**Oligonucleotides**		
qPCR primer sequences	This study	Supplementary Table S1
**Software and algorithms**		
GraphPad Prism 9 (analysis and graphing)	GraphPad Software	RRID:SCR_002798
CFX Maestro (qPCR data analysis software)	Bio-Rad Laboratories	N/A
WormLab (*C. elegans* tracking software)	MBF Bioscience	RRID:SCR_017669
IMT i-Solution (imaging analysis software)	IMT i-Solution Inc.	N/A
**Other**		
Olympus IX53 inverted fluorescence microscope	Olympus Corp.	RRID:SCR_015801
Bio-Rad CFX96 Touch Real-Time PCR System	Bio-Rad Laboratories	RRID:SCR_018064
Qubit 4 Fluorometer (RNA quantification)	Invitrogen	RRID:SCR_018093
Illumina TruSeq Stranded Total RNA Library Prep Gold Kit	Illumina	Cat #20020599
SuperScript II reverse transcriptase	Invitrogen	Cat #18064014
NovaSeq6000	Illumina	RRID:SCR_016387
Field-emission scanning electron microscope (FE-SEM)	Carl Zeiss	SUPRA 55VP
Nexera GPC System	SHIMADZU	RRID:SCR_026529

Table S2. DAF signaling and downstream gene expression in SCGB1-fed worms

Gene name	SCGB1-fed + Mi-PS worms (TPM)	SCGB1-fed worms (TPM)	Fold change (Mi-PS with vs. without)
daf-2	**29.412**	**31.612**	**0.930**
daf-12	**0.743**	**0.723**	**1.028**
daf-16	**9.541**	**9.029**	**1.057**
hsp-12.1	**3.204**	**2.101**	**1.525**
hsp-16.1	**12.844**	**9.893**	**1.298**
hsp-16.2	**8.359**	**5.298**	**1.578**
hsp-70	**2.056**	**1.558**	**1.320**
pha-4	**0.716**	**0.709**	**1.010**
lgg-1	**1104.641**	**970.483**	**1.138**
hlh-30	**3.471**	**3.131**	**1.109**
ctl-1	**45.674**	**43.409**	**1.052**
ctl-2	**42.345**	**43.609**	**0.971**
sod-3	**2.836**	**1.818**	**1.560**

Supplementary Texts

Text S1. Preparation of biofilm-induced bacterial biomass using 25 μm microparticles

To generate biofilm-induced biomass, 25 μm polystyrene microspheres (Mi-PS) and 25 μm silica microspheres (Mi-Si) were prepared and incorporated into NGM medium at 0.1% (w/v). Bacterial cultures were inoculated onto the microparticle-containing NGM to a final cell-mass concentration of 0.03 g/mL and incubated for an additional 50 h to allow biofilm formation. Biofilm formation on the microparticles was confirmed prior to biomass harvest (**Fig. S3**).

After incubation, the biofilm-coated microparticles and surrounding culture were collected and subjected to sequential size-based removal of microparticles. First, the suspension was passed through a sterilized 40 μm cell strainer (CLS431750-50EA , CORNING) using a sterile loop to separate bacterial biomass from the 25 μm microparticles. The filtrate was then passed through a sterilized 5 μm syringe filter (6784-1350, CYTIVA WHATMAN) as a second filtration step to remove any remaining microparticles. The resulting filtrate, containing biofilm-induced bacterial cell mass, was pelleted by centrifugation.

The bacterial pellet was resuspended in M9 buffer supplemented with 10% glycerol, adjusting the suspension to 0.03 g/mL. The washed biomass was stored at −80°C until use. Immediately before experiments, frozen aliquots were thawed and washed twice with M9 buffer to remove glycerol. The final biomass was applied onto NGM agar plates to form bacterial lawns for downstream assays.

Text S2. M9 buffer and Modified Nematode Growth Medium (NGM)

M9 buffer was prepared using the following composition per liter of distilled water: 3 g KH_2PO_4, 6 g Na_2HPO_4, 5 g NaCl, and 1 mL of 1 M $MgSO_4$ [1]. The solution was sterilized by autoclaving and stored at room temperature until use. When suppression of bacterial growth was required, an antibacterial variant of M9 buffer was prepared by supplementing ampicillin (Sigma-Aldrich, A9518, USA) and streptomycin (Sigma-Aldrich, S6501, USA) at final concentrations of 100 μg/mL each [2].

Modified NGM was prepared using the following composition per liter of distilled water: 3 g NaCl, 3.5 g peptone, and 20 g agar [3]. After autoclaving, the medium was supplemented with 1 mL of 1 M $MgSO_4$, 1 mL of 1 M $CaCl_2$, 1 mL of 5 mg/mL cholesterol in ethanol, 25 mL of 1 M KPO_4 buffer, and 5 mL of Nystatin suspension (Sigma-Aldrich, N1638, USA; final concentration, 50 U/mL). When suppression of bacterial growth was required, an antibacterial variant of NGM was prepared by supplementing the medium with ampicillin (Sigma-Aldrich, A9518, USA) and streptomycin (Sigma-Aldrich, S6501, USA) at final concentrations of 100 μg/mL each [2].

Text S3. Standardization of bacterial cell mass

To obtain identical bacterial biomass for *C. elegans* exposure, all strains were cultured in Luria–Bertani (LB) broth. For cultivation, a single bacterial colony was inoculated and incubated aerobically at 37°C with shaking at 180 rpm for 24 h. Following incubation, cultures were washed and resuspended in M9 buffer to standardize biomass at approximately 0.03 g/mL (equivalent to 30,000 mg/L, wet cell mass). Under these standardized conditions, the corresponding OD_{600} values and colony-forming units (CFU/mL) were:

• *E. hormaechei* LG3: OD_{600} = 1.887 (8.3 × 10^9 CFU/mL)
• *B. amyloliquefaciens* SCGB1: OD_{600} = 1.811 (2.1 × 10^9 CFU/mL)
• *E. coli* OP50: OD_{600} = 1.866 (6.5 × 10^9 CFU/mL)

Text S4. Plate-based co-exposure (Mi-PS + Bacteria)

Bacterial suspensions (30,000 mg/L in M9 buffer) were combined with serially diluted Mi-PS (polystyrene microsphere) in M9 buffer at a 100:1 ratio, yielding final Mi-PS concentrations of 0, 1, and 10 mg/L (w/v). To minimize potential oxidation of Mi-PS caused by contact with plastic-biodegrading bacteria, the bacterial suspensions and Mi-PS were mixed immediately before exposure experiments [4, 5]. A 100-μL aliquot of the Mi-PS–bacteria mixture was evenly spread on NGM agar in a 2 cm × 2 cm square within 35-mm Petri dishes to generate exposure lawns (**Fig. S5**). Subsequently, synchronized *C. elegans* were placed on these prepared exposure lawns to allow co-exposure and feeding on the bacteria and Mi-PS mixtures [6].

Text S5. Field-emission scanning electron microscopy (FE-SEM) and Fluorescence Microscopy

- **Field emission scanning electron microscope (FE-SEM)**

Specimen surface morphology was examined using a field-emission scanning electron microscope (SIGMA, Carl Zeiss, Germany) operated at 5 kV accelerating voltage. The instrument provides 1.0 nm resolution at 1.5 kV emission voltage. Images were acquired at 100,000× magnification. Prior to imaging, samples were sputter-coated with a thin platinum layer to improve conductivity and minimize charging.

- **Fluorescence microscopy and quantification of Mi-PS capture in biofilms**

For each microbial culture, a 2 μL aliquot was placed on a microscope slide to assess Mi-PS attachment within biofilms. Fluorescence images were collected under fixed exposure settings, and the Mi-PS attachment ratio was quantified by examining the entire 2 μL drop using fluorescence microscopy.

$$\text{Mi -PS attachment ratio} = \frac{\text{Mi -PS particles attached to biofilms}}{\text{Total Mi -PS particles}}$$

- **Comparative analysis of carbon-dependent cell growth and Mi-PS attachment**

To compare Mi-PS attachment and bacterial growth under different carbon conditions, we used NGM and liquid carbon-free basal medium (LCFBM; KH_2PO_4 1.5 g/L, K_2HPO_4 1.5 g/L, NH_4NO_3 1.0 g/L, $MgSO_4 \cdot 7H_2O$ 0.2 g/L, $CaCl_2 \cdot 2H_2O$ 0.02 g/L, $FeSO_4 \cdot 7H_2O$ 0.001 g/L; pH 7.0). Media (20 mL) were aliquoted into 240 mL Wheaton^{TM} glass serum bottles (844082; DWK Life Sciences). Bacterial cultures grown in LB were washed three times with M9 buffer, and 200 µL of the washed suspension was inoculated into each bottle. After inoculation, bottles were vortexed for 10 s. An initial 100 µL aliquot was collected to quantify baseline viable counts by serial dilution and plating on LB agar for CFU enumeration. Cultures were then incubated at 25°C with shaking at 180 rpm. After 48 h, cultures were sampled and the Mi-PS attachment ratio was determined by fluorescence microscopy.

Text S6. Solid-phase microextraction gas chromatography–mass spectrometry (SPME/GC–MS) analysis

Cultured samples were subjected to SPME/GC–MS to identify and quantify low-carbon-backbone and aromatic species [4, 7]. SPME/GC–MS analysis was performed using a Thermo TRACE 1310/TSQ 8000 GC–MS system equipped with a DB-Wax capillary column (122–7063, Agilent, USA). Pure helium (99.999%) was used as the carrier gas. The GC parameters were as follows: 2 mL of sample was injected into the inlet at 250°C in split mode (10:1) at a flow rate of 20 mL/min. The oven temperature was programmed as follows: hold at 40°C for 2 min, ramp to 150°C at 4°C/min, hold for 10 min, then increase to 200 °C at 4°C/min and hold for 5 min, followed by a final ramp to 230°C at 4°C/min and a 5 min hold. 1,2-Dichlorobenzene-d4 was used as the internal standard for quantitative analysis. Spectral peaks were identified by comparison against the NIST/EPA/NIH Mass Spectral Library (version 2.4g) using the NIST Mass Spectral Search Program. Metabolites were filtered by requiring greater peak areas than the blank control and library-match scores of SI ≥ 700 (**Fig. S6**).

Text S7. Surface chemistry and molecular weight of Mi-PS

- Bacterial-treated Mi-PS sample preparation.

Cultured samples were centrifuged at 10,000 rpm for 10 min. The pellets were treated with 5 mL of 6 N HCl and incubated at room temperature for 48 h [8]. After adding 10 mL of M9 buffer, the samples were neutralized by adding 5 mL of 6 N NaOH and allowing the mixture to stabilize for 30 min. The tubes were inverted 10

times, centrifuged again at 10,000 rpm for 10 min, and the supernatant was discarded. The pellets were washed twice with distilled water under the same centrifugation conditions. Next, 10 mL of 2% (w/v) sodium dodecyl sulfate (SDS) solution was added, and the mixture was rotated at 6 rpm at room temperature for 1 h [9]. After centrifugation at 10,000 rpm for 10 min, the supernatant was removed, leaving a small residual volume above the pellet. The remaining suspension was homogenized by pipetting, and 1 mL was transferred to a new 2 mL microtube. Final washing was performed three times with 80% ethanol (13,500 rpm, 5 min each), followed by drying at 40°C for 24 h.

- Fourier transform infrared spectroscopy (FTIR) analysis.

The surface chemistry of the treated Mi-PS beads was characterized by FTIR. For “initial time” samples, bacterial strains and Mi-PS beads were mixed and immediately subjected to the HCl treatment step (without incubation), followed by the same downstream processing.

- Gel permeation chromatography (GPC) analysis.

For each GPC sample, 10 mg of Mi-PS was collected and dissolved in tetrahydrofuran (THF). The weight-average molecular weight (M_w) and number-average molecular weight (M_n) of THF-dissolved Mi-PS were determined by GPC.

Text S8. *C. elegans* lifespan assays

- Lifespan analysis with live bacterial cell mass.

Lifespan assays were performed using the AU37 strain of *C. elegans*. To ensure consistent developmental timing, synchronized L1 larvae were initially cultured on NGM plates (35 mm) seeded with *E. coli* OP50 at 25°C for 40 h until reaching the L4 stage, as feeding different bacterial strains from the L1 stage can alter the time required to reach adulthood [10]. The synchronized L4 worms were collected with 10 mL of M9 buffer, transferred to 15 mL conical tubes, and washed three times with M9 buffer to a final volume of 14 mL. Worms were then transferred to NGM plates containing lawns of either *E. coli* OP50, *E. hormaechei* LG3, or *B. amyloliquefaciens* SCGB1, each prepared without Mi-PS or Mi-Si. Subsequently, 30 worms were transferred to fresh NGM plates seeded with the same bacterial strains supplemented with Mi-PS or Mi-Si (silica microsphere; 0, 1, or 10 mg/L). Each condition was conducted in triplicate.

Worm viability was assessed every 12 h by manual scoring, and dead individuals were removed at each time point. Every 48 h, worms were transferred to fresh NGM plates containing newly prepared bacterial lawns with the corresponding particle treatment. The time point of the last surviving worm was recorded as the maximum lifespan, and survival data were used to calculate lifespan for each group.

- Lifespan analysis with heat-killed bacterial cell mass.

Lifespan assays were performed using the AU37 strain of *C. elegans* to evaluate the impact of heat-killed bacterial mass on worm longevity. Bacterial suspensions (0.03 g/mL) were autoclaved at 121°C for 15 min to prepare heat-killed cells. Subsequently, 100 µL of each autoclaved suspension was seeded onto modified NGM plates containing ampicillin and streptomycin (100 µg/mL each) to prevent residual microbial growth. To ensure consistent developmental timing, synchronized L1 larvae were initially cultured on NGM plates seeded with live *E. coli* OP50 at 25°C for 40 h until reaching the L4 stage, as exclusive feeding of heat-killed bacteria from the L1 stage does not provide sufficient nutrition for proper development to adulthood [11]. Prior to exposure, synchronized L4 worms were collected with 10 mL of antibiotic-supplemented M9 buffer (100 µg/mL each of ampicillin and streptomycin), transferred to 15 mL conical tubes, and washed three times with the same antibiotic-supplemented M9 buffer to a final volume of 14 mL [12]. The worms were then gently rotated for 1 h at 6 rpm using a rotator (SLB, SLRM-3 Intelli-Mixer, South Korea).

Following preparation, the synchronized L4 worms were transferred to NGM plates containing lawns of heat-killed *E. coli* OP50, *E. hormaechei* LG3, or *B. amyloliquefaciens* SCGB1, each prepared without Mi-PS. Subsequently, 30 worms were transferred to antibiotic-supplemented NGM plates seeded with the same heat-killed bacterial strains supplemented with Mi-PS (0 or 10 mg/L). Each condition was conducted in triplicate. Worm viability was monitored every 12 h by manually scoring and removing dead individuals. Worms were transferred daily to fresh antibiotic-supplemented NGM plates containing newly prepared bacterial lawns with Mi-PS, as the bacterial mass was rapidly consumed. The time point of the last surviving worm was recorded as the maximum lifespan, and daily survival data were used to calculate lifespan for each group.

- Lifespan analysis with biofilm-induced biomass.

Immediately before assays, biofilm-induced biomass prepared as described in **Text S1** was thawed at room temperature, washed with M9 buffer, and a 100-µL aliquot was inoculated onto NGM plates to establish bacterial lawns for lifespan analysis. The lifespan assay procedure (worm synchronization, transfer scheme, scoring frequency, and plate renewal) followed the protocol described for **Lifespan analysis with live bacterial cell mass**.

- Lifespan analysis using biomass supplemented with volatile metabolites.

Stock solutions of isoamyl alcohol (IAA), isobutyrate (IBA), and isovalerate (IVA) were prepared in M9 buffer at 100 mM. The pH was adjusted to 7.0 with 5 N NaOH, and the solutions were sterilized by filtration (0.22 µm). For each condition, 10 mL working solutions (10 mM) were prepared, and Mi-PS were added to a final concentration of 10 mg/L.

An *E. coli* OP50 culture was collected, washed twice with M9 buffer, and adjusted to 0.03 g/mL. For conditioning, 1 mL of the OP50 suspension was transferred to a 2-mL microtube, centrifuged, and the supernatant was removed. The pellet was resuspended in 10 mM IAA, IBA, or IVA (each in M9 buffer containing 10 mg/L Mi-PS) or in blank M9 buffer (0 mM; containing 10 mg/L Mi-PS) as a control. Aliquots (100 μL) of the volatile metabolite–supplemented biomass were spread onto 30-mm NGM agar plates. The subsequent lifespan assay procedure followed the protocol described for "**Lifespan analysis with live bacterial cell mass**".

- Lifespan analysis using microbially pre-treated Mi-PS.

Bacterial-pretreated Mi-PS was recovered following the "**Bacterial-treated Mi-PS sample preparation**" procedure described above. Before the final drying step, at the stage immediately prior to transferring the suspension to a new 2 mL microtube, the empty microtube was pre-weighed using an analytical microbalance (PX224KR, OHAUS). After transfer, the tube was weighed again to determine the mass of Mi-PS in the suspension by difference. Next, 1 mL of M9 buffer was added, and the mixture was vortexed for 5 min. Any remaining particle aggregates were further dispersed by repeated pipetting. The suspension was then adjusted to a 1 g/L Mi-PS stock solution. Subsequent lifespan assays were performed as described in the "**Lifespan analysis with live bacterial cell mass**" protocol.

Text S9. *C. elegans* oxidative stress analysis

Oxidative stress was assessed in the AU37 strain of *C. elegans*. Worms were exposed either to bacteria alone (*E. coli* OP50, *E. hormaechei* LG3, or *B. amyloliquefaciens* SCGB1) or to the same bacteria mixed with Mi-PS (10 mg/L). Synchronized L1 larvae were cultured on NGM plates seeded with *E. coli* OP50 at 25°C for 40 h until reaching the L4 stage. Worms were then collected in 10 mL of M9 buffer, transferred to 15 mL conical tubes, and washed three times with M9 buffer to a final volume of 14 mL. L4 worms were then transferred to NGM plates containing lawns of *E. coli* OP50, *E. hormaechei* LG3, or *B. amyloliquefaciens* SCGB1 prepared without Mi-PS. Subsequently, 30 worms per plate were transferred to fresh NGM plates seeded with the same bacterial strains, either without Mi-PS or with 10 mg/L Mi-PS. The worms were incubated at 25°C for 50 h, and dead individuals were excluded from the analysis.

Reactive oxygen species (ROS) were detected using the fluorescent probe CM-H_2DCFDA (C6827, Invitrogen). A working solution of 15 μM CM-H_2DCFDA in M9 buffer was prepared. For staining, 2 mL of worms in M9 buffer was mixed with 4 mL of the dye solution and incubated in the dark at 25°C for 120 min [13]. After staining, worms were anesthetized with 0.03 M sodium azide in M9 buffer and mounted on glass slides. Fluorescence was visualized using a microscope (IX53, Olympus, Japan) light-restricted conditions using standardized imaging parameters in i-Solution software (IMT i-Solution, Korea): histogram target level of 100, exposure time of 14.701 ms, and gain of 4.59. Fluorescence intensity and area were quantified using i-Solution software.

Text S10. ***C. elegans*** **growth rate and locomotion analysis**

Synchronized L1 worms (30 per plate) were cultured on bacterial lawns (*E. coli* OP50, *E. hormaechei* LG3, or *B. amyloliquefaciens* SCGB1) mixed with Mi-PS at concentrations of 0, 1, or 10 mg/L for 50 or 100 h. All experiments were conducted in triplicate. Worms were then washed with M9 buffer and transferred to fresh NGM plates without bacterial lawns for imaging. After a 20 min acclimation period, locomotor behavior was recorded for 30 s using a stereomicroscope (SMZ745T, Nikon, Japan) at 6.7× magnification. Each plate was imaged in three separate sections, resulting in nine recordings per group. Worms that overlapped or moved out of the field of view during imaging were excluded from analysis. Worm length, width, and locomotion speed were quantified using WormLab software (MBF Bioscience, USA) with a threshold level of 85 and hotspot illumination correction.

Text S11. Quantification of luminally accumulated Mi-PS in ***C. elegans***

- Quantification of luminally accumulated Mi-PS using live bacterial cell mass.

Luminal localization of Mi-PS was assessed in the AU37 strain of *C. elegans* to evaluate strain-specific differences. Subtle differences in host developmental rate have been observed in *C. elegans* depending on the bacterial diet provided (**Fig. S7**). Most somatic growth in *C. elegans* occurs during the larval stages (L1–L4), with body size largely stabilized upon entry into adulthood [14]. pharyngeal pumping rates vary across larval development and increase slightly from L1 to L4 [15], which can increase particle ingestion, and because body size can influence intestinal residence time and thus retention, we standardized the developmental stage at exposure initiation. Specifically, synchronized L1 larvae were cultured on NGM plates seeded with E. coli OP50 at 25°C for 40 h until reaching the L4 stage (**Fig. S7A**)

L4 worms were then transferred to NGM plates containing lawns of *E. coli* OP50, *E. hormaechei* LG3, or *B. amyloliquefaciens* SCGB1, each prepared without Mi-PS. Subsequently, 30 worms per plate were transferred to fresh NGM plates seeded with the same bacterial strains supplemented with 10 mg/L Mi-PS, with transfers to freshly prepared plates every 48 h. All experiments were performed in triplicate.

After 50 or 100 h of incubation at 25°C, worms were transferred to fresh plates containing the same bacterial lawns but without Mi-PS and incubated for 6 h, followed by four washes with M9 buffer, performed by sequentially transferring worms between fresh 35 mm Petri dish containing M9 buffer, and an additional 10 h incubation in fresh M9 buffer at room temperature to ensure clearance of unretained particles. Dead individuals were excluded from analysis. Sixteen worms per plate were randomly selected and transferred to individual wells of a 96-well plate, and the number of luminally accumulated Mi-PS was performed using fluorescence microscopy.

- Quantification of luminally accumulated Mi-PS using heat-killed bacterial cell mass.

To examine the effect of bacterial viability on Mi-PS accumulation, AU37 worms were cultured and synchronized to L4 stage. After four washes with M9 buffer, worms were incubated in antibiotic-supplemented M9 buffer (100 μg/mL ampicillin and streptomycin) in 15 mL conical tubes for 1 h at room temperature with gentle rotation (6 rpm) to eliminate residual live bacteria [12]. L4 worms were then transferred to antibiotic-supplemented NGM plates containing heat-killed lawns of *E. coli* OP50, *E. hormaechei* LG3, or *B. amyloliquefaciens* SCGB1 without Mi-PS. Thirty worms per plate were subsequently transferred to fresh plates seeded with the same heat-killed strains supplemented with 10 mg/L Mi-PS, with transfers every 24 h. After 50 h of incubation at 25°C, worms were moved to plates containing the same heat-killed lawns without Mi-PS and incubated for 6 h, followed by post-exposure clearance and washing with antibiotic-supplemented M9 buffer. Quantification of luminally accumulated Mi-PS was performed using fluorescence microscopy.

- Quantification of luminally accumulated Mi-PS in DAF pathway mutants.

To investigate the role of the DAF signaling pathway in Mi-PS accumulation, comparative assays were performed using four *C. elegans* strains: N2 (wild-type), GR1307 [*daf-16*(mgDf50) I], AA1 [*daf-12*(rh257) X], and AMH55 [*daf-2*(e1370) III; *otIs117* IV]. Synchronized L1 larvae were cultured on SCGB1-seeded NGM plates at 20°C for 80 h until adulthood, as SCGB1 delayed maturation relative to other bacterial strains. NGM plates were supplemented with 80 mM FUdR to suppress reproduction during the experimental period. Adult worms were then transferred to plates containing SCGB1 lawns without Mi-PS, followed by transfer of 30 worms per plate to fresh plates seeded with SCGB1 supplemented with 10 mg/L Mi-PS, with transfers every 48 h. After 20 or 60 h of incubation at 25°C, worms were moved to plates containing SCGB1 lawns without Mi-PS and incubated for 6 h, followed by post-exposure clearance and washing with M9 buffer. Quantification of luminally accumulated Mi-PS was performed using fluorescence microscopy.

- Quantification of luminally accumulated Mi-PS under dose-dependent LPS exposure.

To assess the effect of LPS on the host response, *C. elegans* were exposed to purified LPS (Sigma-Aldrich, L4391) derived from *E. coli* O111:B4, which is widely used as a standard LPS preparation [16]. Synchronized L1 larvae were cultured on NGM plates seeded with *E. hormaechei* LG3 at 25°C for 40 h until reaching the L4 stage. To minimize co-transferred bacterial biomass (including LPS-containing components), worms were first suspended from the bacterial lawn using M9 buffer and then washed by sequentially transferring them between fresh 35 mm Petri dishes containing antibiotic-supplemented M9 buffer (100 μg/mL each of ampicillin and streptomycin). Washed worms were subsequently collected in 10 mL of antibiotic-supplemented M9 buffer, transferred to 15 mL

conical tubes, and washed four additional times (2,500 rpm, 1 min each). Following these washes, worms were incubated in antibiotic-supplemented M9 buffer for 1 h at room temperature with gentle rotation (6 rpm) to eliminate residual live bacteria [12].

For exposure, 30 worms per plate were transferred to fresh NGM plates without bacterial lawns. The plates were air-dried in a clean bench for 20 min to allow evaporation of the M9 buffer. Then, 100 µL of antibiotic-supplemented M9 buffer containing Mi-PS (10 mg/L) and either LPS (0, 1, 10 or 100 µg/mL) was applied to the center of each plate to form exposure lawns. Plates were immediately sealed, wrapped with parafilm, and incubated at 25°C for 20 h.

After incubation, worms were transferred to fresh plates containing heat-killed LG3 lawns without Mi-PS and incubated for 6 h. Post-exposure clearance included four sequential washes with fresh M9 buffer in 35 mm Petri dishes and an additional 10 h incubation in fresh M9 buffer at room temperature to ensure clearance of unretained particles. Dead individuals were excluded from analysis. Sixteen worms per plate were randomly selected and transferred to individual wells of a 96-well plate, and the number of luminally localized Mi-PS particles was quantified using a fluorescence microscope.

In the absence of bacterial biomass, exposure frequency to Mi-PS increased, leading to substantial luminal accumulation of Mi-PS. Because this accumulation hindered the accurate counting of individual particles, the total fluorescent area of Mi-PS was quantified instead (**Fig. S12**). Imaging of individual worms was performed under light-restricted conditions using standardized parameters in i-Solution software (IMT i-Solution, Korea; histogram target level = 70, exposure time = 23.778 ms, gain = 1.00). Images were converted to 8-bit, background-subtracted (rolling ball radius = 50 px), and binarized using a fixed intensity threshold of 6 (0–255). The same threshold was applied to all samples. Total fluorescent area per field was quantified from the resulting masks using the Threshold tool.

- Quantification of luminally accumulated Mi-PS using biomass supplemented with volatile metabolites.

Stock solutions of IAA, IBA, and IVA were prepared in M9 buffer at 100 mM. The pH was adjusted to 7.0 with 5 N NaOH, and the solutions were sterilized by filtration (0.22 µm). For each condition, 10 mL working solutions (10 mM) were prepared, and Mi-PS were added to a final concentration of 10 mg/L.

An *E. coli* OP50 culture was collected, washed twice with M9 buffer, and adjusted to 0.03 g/mL. For conditioning, 1 mL of the OP50 suspension was transferred to a 2-mL microtube, centrifuged, and the supernatant was removed. The pellet was resuspended in 10 mM IAA, IBA, or IVA (each in M9 buffer containing 10 mg/L Mi-PS) or in blank M9 buffer (0 mM; containing 10 mg/L Mi-PS) as a control. Aliquots (100 µL) of the volatile metabolite–supplemented biomass were spread onto 30-mm NGM agar plates. The subsequent lifespan assay procedure followed the protocol described for **Quantification of luminally accumulated Mi-PS using live bacterial cell mass**.

Text S12. Quantification of DAF-12 and DAF-16 GFP nuclear localization

To assess host responses to volatile metabolites, we quantified nuclear localization of DAF-12::GFP and DAF-16::GFP following exposure to IAA, IBA, and IVA (**Fig. S13**). Stock solutions of IAA, IBA, and IVA were prepared in NGM liquid medium at 100 mM, adjusted to pH 7.0 with 5 N NaOH, and filter-sterilized (0.22 μm). Working solutions were prepared in NGM liquid medium at final concentrations of 0, 1, and 10 mM for each metabolite.

C. elegans strains DAF-12::GFP (OH14589) and DAF-16::GFP (MQD1543) were maintained on NGM agar plates seeded with *E. coli* OP50 and synchronized to the L4 stage. L4 animals were collected and washed three times with NGM liquid medium containing the corresponding metabolite concentration (0, 1, or 10 mM). Worms were then exposed for 1 h in NGM liquid medium containing IAA, IBA, or IVA (0, 1, or 10 mM). After exposure, animals were immobilized and fixed with paraformaldehyde (PFA) (4% stock; final concentration 1%) and imaged. All imaging was completed within 10 h of fixation. For each independent experiment, 10–30 animals were analyzed per group, and experiments were performed in triplicate. Fluorescence imaging was performed under light-restricted conditions using an IX53 microscope with i-Solution software using standardized acquisition settings (histogram target level, 100; exposure time, 14.701 ms; gain, 4.59). Nuclear localization was scored based on the presence or absence of nuclear puncta, and the proportion of animals exhibiting nuclear puncta was quantified [17, 18].

Text S13. Assessment of endotoxin release by Gram-negative bacteria in the presence of Mi-PS

To quantify endotoxin release from Gram-negative bacteria in the presence of Mi-PS, *E. hormaechei* LG3 and *E. coli* OP50 were incubated with Mi-PS (0 mg/L or 10 mg/L) on NGM plates under aerobic conditions at 25°C for 50 h. After incubation, bacterial biomass was collected using a 1 μL inoculation loop and suspended in 1 mL of 0.1% Triton X-100 solution [19, 20]. The suspension was vortexed for 10 min, incubated overnight at 4°C, and homogenized by pipetting. The optical density at 600 nm (OD_{600}) was measured, and suspensions were adjusted to comparable levels (OD_{600} ± 0.010). Endotoxin concentrations were determined using the ToxinSensor™ Chromogenic LAL Endotoxin Assay Kit (GenScript, Cat. No. L00350) according to the manufacturer's instructions and quantified using a standard curve (**Fig. S14**).

Text S14. Transcriptomics and qPCR

- Total cDNA analysis of *C. elegans.*

Total cDNA analysis was performed using the AU37 strain of *C. elegans* to assess gene expression changes. Synchronized L1 worms (200 individuals per 90 mm Petri dish) were cultured on bacterial lawns (E. coli OP50, *E. hormaechei* LG3, or *B. amyloliquefaciens* SCGB1) supplemented with Mi-PS at final concentrations of 0 or 10 mg/L for 100 h at 25°C. Six experimental conditions were established according to bacterial strain and Mi-PS exposure, and each condition was performed in

duplicate. Worms were transferred to fresh NGM plates every 48 h, and dead individuals were manually removed throughout incubation.

To minimize carryover of bacterial biomass prior to RNA preparation, worms were suspended from the bacterial lawns in M9 buffer and sequentially washed by transferring them to fresh 90 mm Petri dishes containing M9 buffer. From each group, 100 worms were randomly collected from two plates (200 worms selected per experimental conditions) and preserved in TRIzol™ for RNA extraction [21, 22]. Total RNA was purified using the RNeasy Mini Kit, quantified with RiboGreen®, and assessed for integrity using the Agilent TapeStation. Only samples with RNA integrity numbers (RIN) > 7.0 were used for downstream analysis.

RNA-seq libraries were prepared from 0.5 μg of total RNA using the TruSeq Stranded Total RNA Library Prep Kit. Following rRNA depletion and fragmentation, cDNA was synthesized, end-repaired, A-tailed, adapter-ligated, and PCR-amplified. Library quality and concentration were confirmed prior to paired-end sequencing (2 × 100 bp) on the Illumina NovaSeq 6000 platform (Illumina Inc., USA).

Raw reads were quality-trimmed using Trimmomatic and aligned to the *C. elegans* reference genome (WBcel235) using HISAT2. Aligned reads were sorted with SAMtools and assembled with StringTie to obtain gene- and transcript-level expression values (raw counts, FPKM, and TPM). Differential expression analysis was performed using edgeR with TMM normalization [23]. The dispersion estimates were inferred from the overall count distribution, and differentially expressed genes were identified primarily based on a fold-change threshold (|fold change| ≥ 2). Nominal p-values and FDR-adjusted values provided by edgeR were interpreted in an exploratory manner rather than as definitive indicators of significance [24]. Functional enrichment analysis of fold-change–filtered genes was performed using gProfiler for GO terms and an in-house KEGG annotation pipeline, with multiple testing correction by the Benjamini–Hochberg method [25, 26]. All analyses were conducted in R version 4.2.2.

- qPCR validation of *fmo-2* expression under Mi-PS + LPS.

qPCR was performed to validate *fmo-2* expression changes in *C. elegans* following exposure to LPS (100 μg/mL) or co-exposure to Mi-PS (10 mg/L) and LPS (100 μg/mL). For exposure, synchronized and washed L4 larvae (200 worms per plate) were transferred onto fresh 90-mm NGM plates without bacterial lawns. Plates were air-dried in a clean bench for 20 min to evaporate residual M9. Subsequently, 500 μL antibiotic-supplemented M9 containing either LPS alone or Mi-PS + LPS was applied to the plate center to form exposure lawns; the blank control received antibiotic-supplemented M9 only. Plates were immediately sealed (parafilm) and incubated at 25°C for 20 h. Dead worms were excluded.

From each group, 150 worms were randomly collected from each of three replicate plates and preserved in TRIzol™ for RNA extraction [21, 22]. cDNA was synthesized using the iScript™ cDNA Synthesis Kit (Bio-Rad, 1708891). qPCR reactions were prepared with RealHelix™ Premier qPCR

Kit [Green, Low ROX] (NanoHelix, PQL-S500) and run on a CFX96 Touch Real-Time PCR Detection System (Bio-Rad, AM07). Primer annealing temperatures were optimized prior to the main run. Cq values were obtained with Bio-Rad CFX Maestro. Primers (Macrogen, Seoul, Korea; 5′→3′) were:

• *fmo2-F*= 5'- AAATCGTTTACTCGTGTTTG -3'
• *fmo2-R* = 5'- TAGTTTTCATAACTGACGAC -3'
• *snb-1-F*= 5'- CCGGATAAGACCATCTTGACG -3'
• *snb-1-R* = 5'- GACGACTTCATCAACCTGAGC -3'

Relative gene expression levels were calculated using the $2^{-\Delta\Delta Ct}$ method [27]. The *snb-1* gene was used as the internal reference for *C. elegans* [28].

● qPCR validation of *lpxA/C/D* expression under Mi-PS + LPS.

qPCR was performed to validate the expression changes of *lpxA*, *lpxC*, and *lpxD* genes in *E. hormaechei* LG3 following exposure to Mi-PS (0, 1, and 10 mg/L). The 100 μL aliquot of each suspension mixed with Mi-PS was spread onto NGM plates to form a lawn. The plates were incubated under aerobic conditions at 25°C for 48 h. After incubation, bacterial lawns from each plate were collected and suspended in TRIzol™ for RNA extraction. Total bacterial RNA was isolated using the RNeasy Mini Kit (Qiagen), and RNA quality and quantity were assessed**.** Primer annealing temperatures were optimized prior to the main run. Cq values were obtained with Bio-Rad CFX Maestro. Primers (Macrogen, Seoul, Korea; 5′→3′) were:

• *lpxD-F*= 5'- AGTTGCGGGTGGTGTTATCA -3'
• *lpxD-R*= 5'- AGCTGTTTTACGCCAGACCT -3'
• *lpxA-F*= 5'- TCGGCGAAGTTAACCAGGAT -3'
• *lpxA-R* = 5'- TAGTTTTCATAACTGACGAC -3'
• *lpxC-F*= 5'- TGTCTGGTGAACGAGCATGA -3'
• *lpxC-R*= 5'- TAAACGAACGGAGCAGCACT -3'
• *rpoA-F*= 5'- ACCGATGAGAACGCAGCTAT -3'
• *rpoA-R*= 5'- TTCTACACGCGCTGCTTCAA -3'

Relative gene expression levels were calculated using the $2^{-\Delta\Delta Ct}$ method. [27] The *rpoA* gene served as the internal reference for *E. hormaechei* LG3 [29].

Supplementary Figures

Fig. S1.

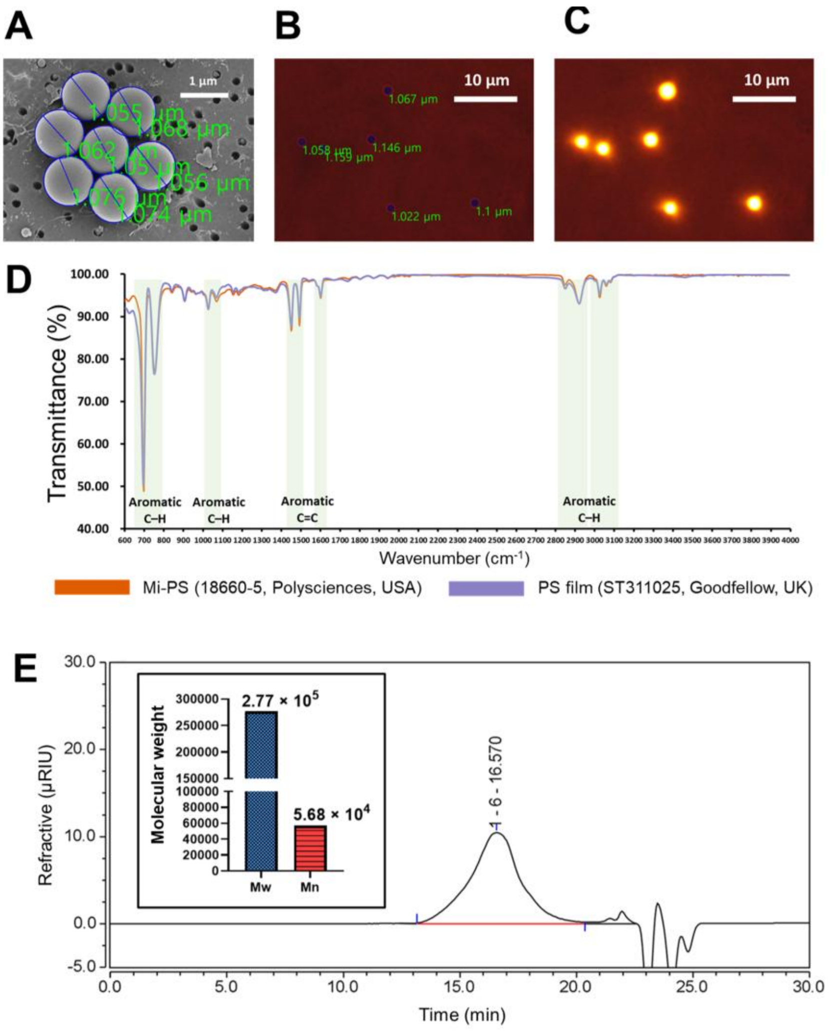

Fig. S1. Morphological and physicochemical characterization of 1-µm Mi-PS. (**A**–**C**) Representative images of 1-µm Mi-PS. (**A**) Scanning electron microscopy (SEM; Zeiss SUPRA 55VP) image showing the spherical morphology of Mi-PS. (**B**–**C**) Bright-field and fluorescence images (Olympus IX53) of the same field; fluorescence was acquired using an excitation wavelength of 491 nm and an emission wavelength of 565 nm to detect the Mi-PS signal. (**D**) FTIR spectra of polystyrene microspheres (Mi-PS; Polysciences, USA) and polystyrene film (PS film; Goodfellow, UK). To assess possible alterations in surface chemistry, PS materials were analyzed by FTIR (Vertex-80 V/Hyperion2000, Bruker Inc.). Spectra were collected from 600 to 4000 cm^{-1} at a resolution of 8 cm^{-1} with 32 scans per sample. Both materials exhibit characteristic aromatic absorption bands, including C–H bending/stretching (~700–1000 cm^{-1}), aromatic C=C stretching (~1450 cm^{-1}), and C–H stretching (~2800–3100 cm^{-1}), confirming the chemical identity of fluorescent Mi-PS relative to the non-labeled PS film. (E) Molecular weight and chromatogram of Mi-PS measured by GPC (Nexera GPC System, SHIMADZU).

Fig. S2.

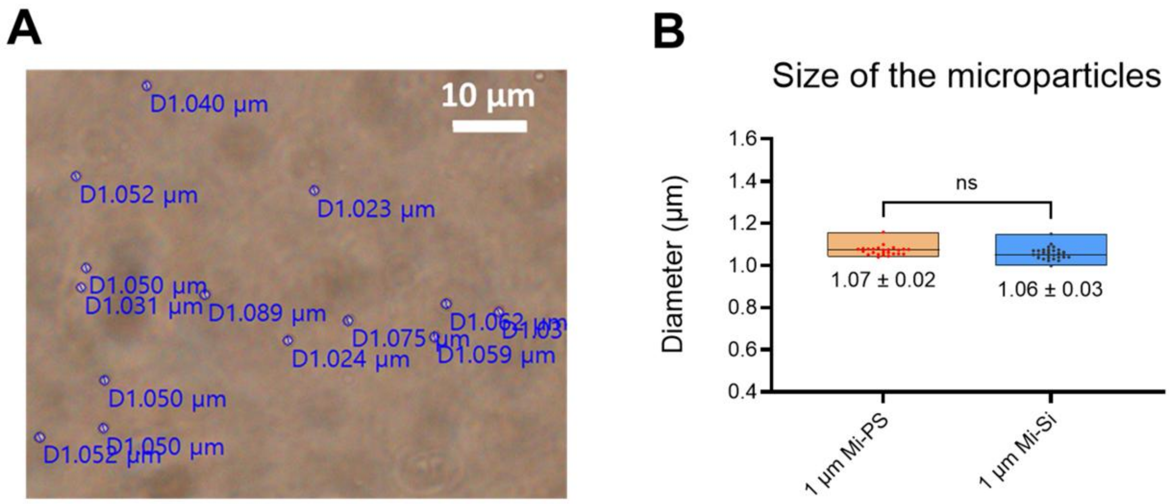

Fig. S2. Morphology- and size-matched silica microspheres (Mi-Si) used as an inert control for Mi-PS. (**A**) Representative bright-field microscopy image of 1-µm silica Mi-Si. (**B**) Quantitative comparison of particle diameter between 1-µm Mi-PS and Mi-Si measured from bright-field images acquired on an Olympus IX53 microscope, showing no significant difference in diameter between the two microparticles.

Fig. S3.

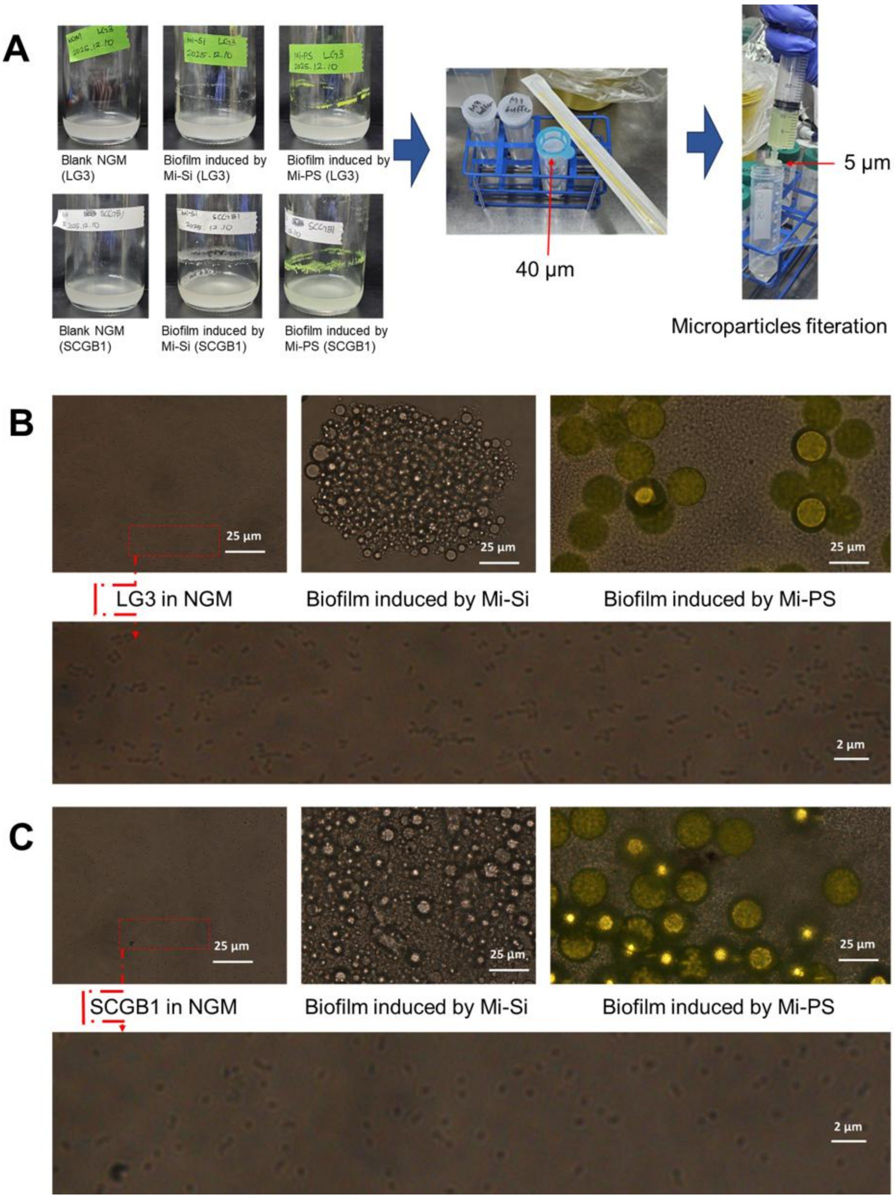

Fig. S3. Workflow for isolating microparticle-induced biofilm–detached cells and representative microscopy of the resulting cultures. (**A**) Schematic of the procedure used to obtain microparticle-induced biofilm–detached cells. Blank NGM contained NGM only, whereas microparticle-supplemented NGM was prepared by adding 25-µm Mi-Si (18241, Polysciences, Inc.) or 25-µm Mi-PS (SiO2-123-50G, GETNANO) to NGM at a final concentration of 0.1% (w/v). After washing with M9 buffer, bacterial biomass was normalized to 0.3 g/mL and incubated under identical aerobic conditions (25°C, 180 rpm) for 48 h. The culture and associated biofilm were then collected and sequentially filtered using a 40-µm cell strainer followed by a 5-µm syringe filter to remove the 25-µm Mi-PS and Mi-Si particles. (**B**–**C**) Representative microscopic images of the cultures, showing (**B**) LG3 and (**C**) SCGB1 under blank NGM, Mi-Si, and Mi-PS conditions.

Fig. S4.

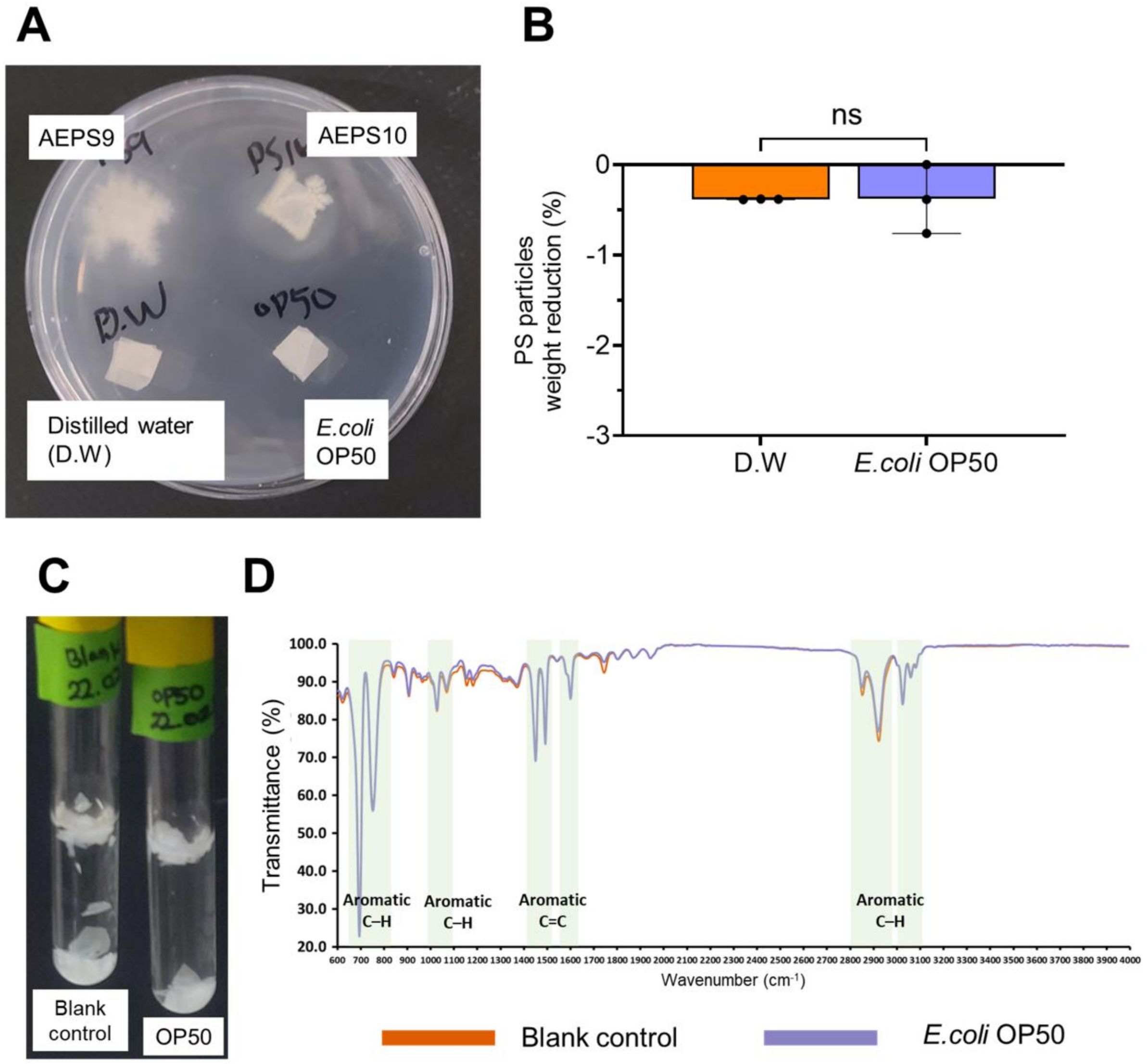

Fig. S4. Non-degradative property of *E. coli* OP50 toward PS. (**A**) Representative plate image of PS films after 20 d incubation in LCFBM (carbon-free basal medium; KH_2PO_4 1.5 g/L, K_2HPO_4 1.5 g/L, NH_4NO_3 1.0 g/L, $MgSO_4 \cdot 7H_2O$ 0.2 g/L, $CaCl_2 \cdot 2H_2O$ 0.02 g/L, $FeSO_4 \cdot 7H_2O$ 0.001 g/L; pH 7.0) containing purified agar (LP0028B, Oxoid) under aerobic conditions at 37°C, inoculated with *E. coli* OP50, candidate PS-degrading strains AEPS9 and AEPS10, or distilled water (negative control) [30]. (**B**) PS film weight reduction after incubation in LCFBM with OP50 compared with the distilled water control; no significant difference was detected. (**C**) Representative culture tubes (10 mL LCFBM) showing PS particles after incubation with E. coli OP50 or the blank control under aerobic conditions (180 rpm) at 37°C. (**D**) FTIR spectra of PS particles recovered from the OP50 and blank control conditions. PS particles collected in (C) were immersed in 2% (w/v) SDS solution for 4 h and then rinsed with 70% (v/v) ethanol, as previously described [4]. FTIR spectra shows no detectable changes in characteristic PS absorption bands, consistent with the absence of chemical modification by OP50.

Fig. S5.

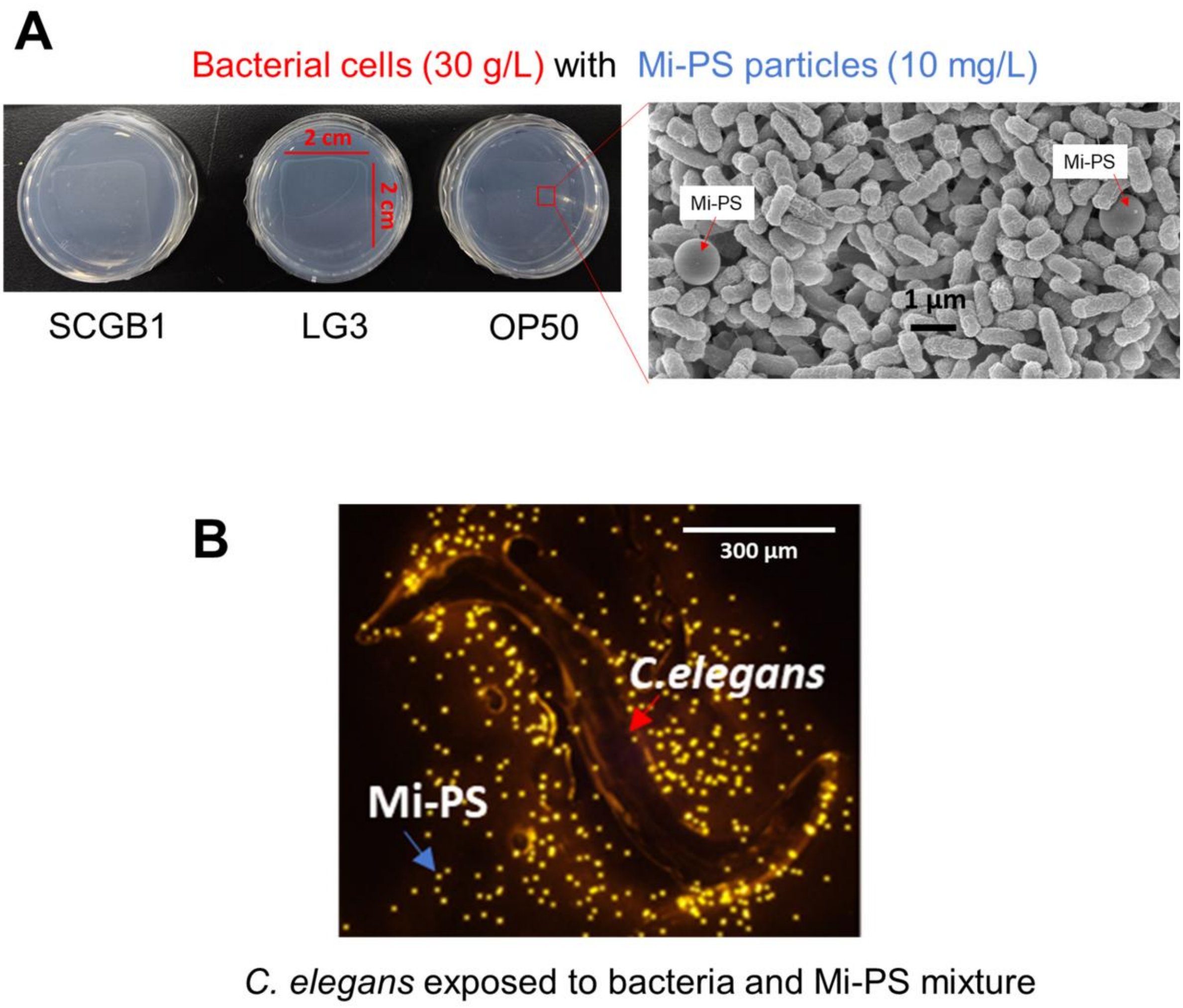

Fig. S5. Experimental setup for co-exposure of *C. elegans* to bacteria and Mi-PS. (**A**) Preparation of exposure lawns on NGM plates containing bacterial biomass (30 g/L wet weight) of *Bacillus amyloliquefaciens* SCGB1, *Enterobacter hormaechei* LG3, or *Escherichia coli* OP50 mixed with 1 µm Mi-PS particles (10 mg/L). A representative SEM image (right) shows Mi-PS particles intermixed with bacterial cells (LG3). (B) Representative fluorescence microscopy image showing *C. elegans* AU37 co-exposed to the bacteria and Mi-PS mixture; yellow, fluorescent signals correspond to Mi-PS particles.

Fig. S6.

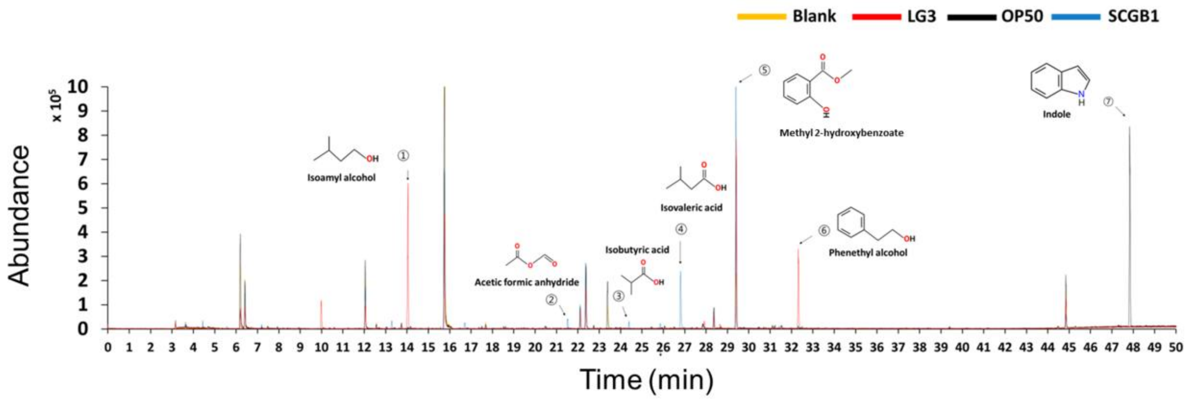

Fig. S6. Representative SPME/GC–MS chromatograms and annotated metabolites. The representative image of chromatograms obtained from bacterial cultures and the blank control in NGM containing Mi-PS 0.1% (w/v). Strain-specific metabolites were annotated by spectral matching to the NIST/EPA/NIH Mass Spectral Library (v2.4g) and were retained when their peak areas were greater than the blank control and the library match score (SI) was ≥ 700.

Fig. S7.

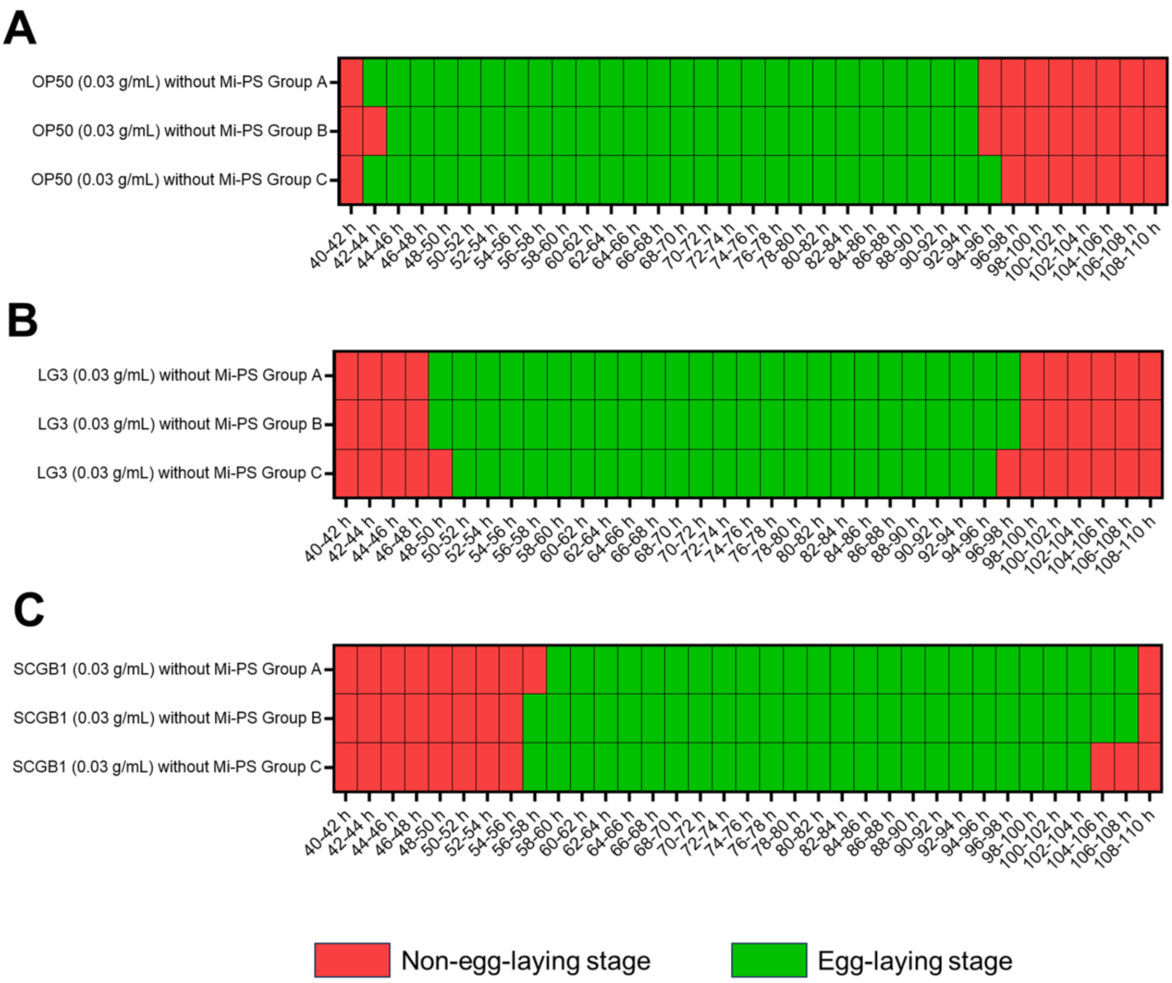

Fig. S7. Adult onset and egg-laying period of *C. elegans* under different bacterial feeding conditions. Heatmap representation of developmental timing and reproductive stage in worms fed (**A**) *E. coli* OP50, (**B**) *B. amyloliquefaciens* SCGB1, or (**C**) *E. hormaechei* LG3 without Mi-PS supplementation. Each row represents one biological replicate group (**A–C**), with 10 worms per replicate, and columns indicate consecutive 2 h intervals from 40 h to 110 h post-hatching.

Fig. S8.

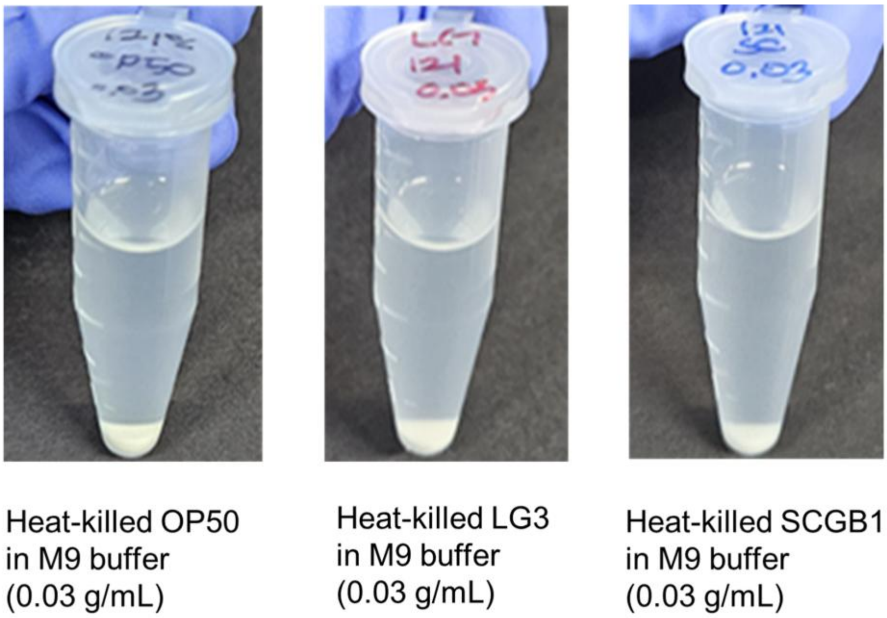

Fig. S8. Preparation of heat-killed bacterial cell mass. Representative images of heat-killed *E. coli* OP50, *E. hormaechei* LG3, and *B. amyloliquefacien*s SCGB1 in M9 buffer at an equal biomass concentration. Bacterial suspensions (0.03 g/mL) were autoclaved at 121°C for 15 min to ensure complete inactivation. Sterilized suspensions were aliquoted (1 mL each) into 1.5 mL microtubes and stored at 4°C. Before use, each tube was vortexed for approximately 5 s to ensure homogeneity.

Fig. S9.

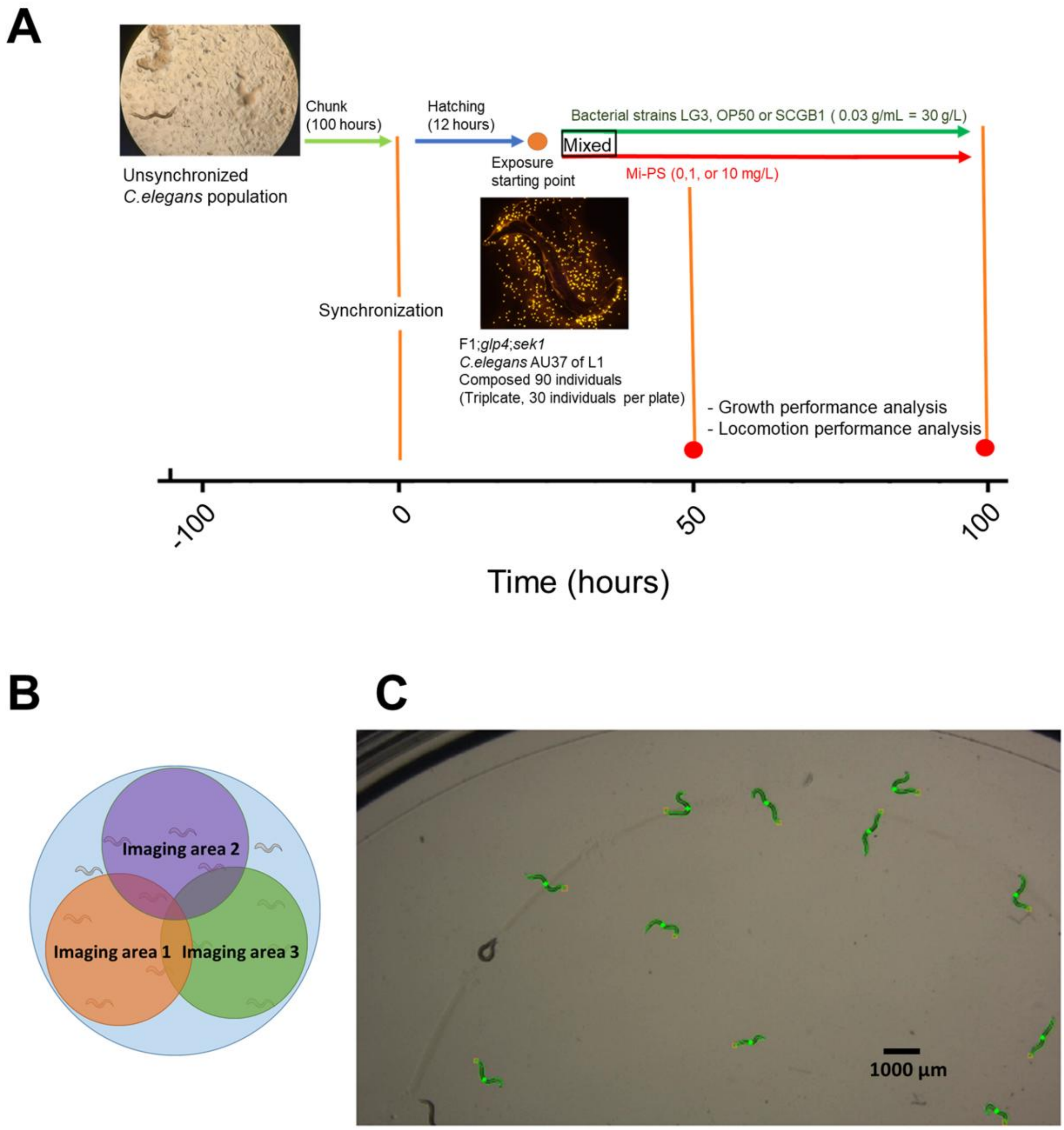

Fig. S9. Workflow and imaging setup for growth and locomotion assays. (A) Experimental timeline. Synchronized L1 larvae (30 worms per plate; triplicate) were cultured on NGM lawns of *E. coli* OP50, *E. hormaechei* LG3, or *B. amyloliquefaciens* SCGB1 mixed with Mi-PS (0, 1, or 10 mg/L) for 50 or 100 h. (**B**) Schematic of the three imaging areas per plate; each plate was recorded in three sections (nine recordings per group). (**C**) Representative image of tracked worms used for morphometric (body length, body width) and locomotion speed analyses. Quantification was performed with WormLab (MBF Bioscience) using threshold 85 and hotspot illumination correction.

Fig. S10.

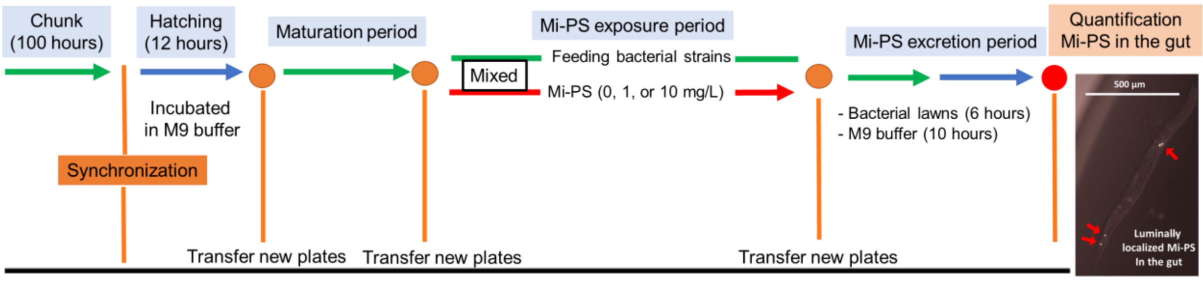

Fig. S10. Workflow for quantification of bioaccumulated Mi-PS in the gut. Schematic of the experimental procedure used to quantitatively assess the amount of Mi-PS that remains retained and accumulates at fixed intestinal locations rather than being expelled.

Fig. S11.

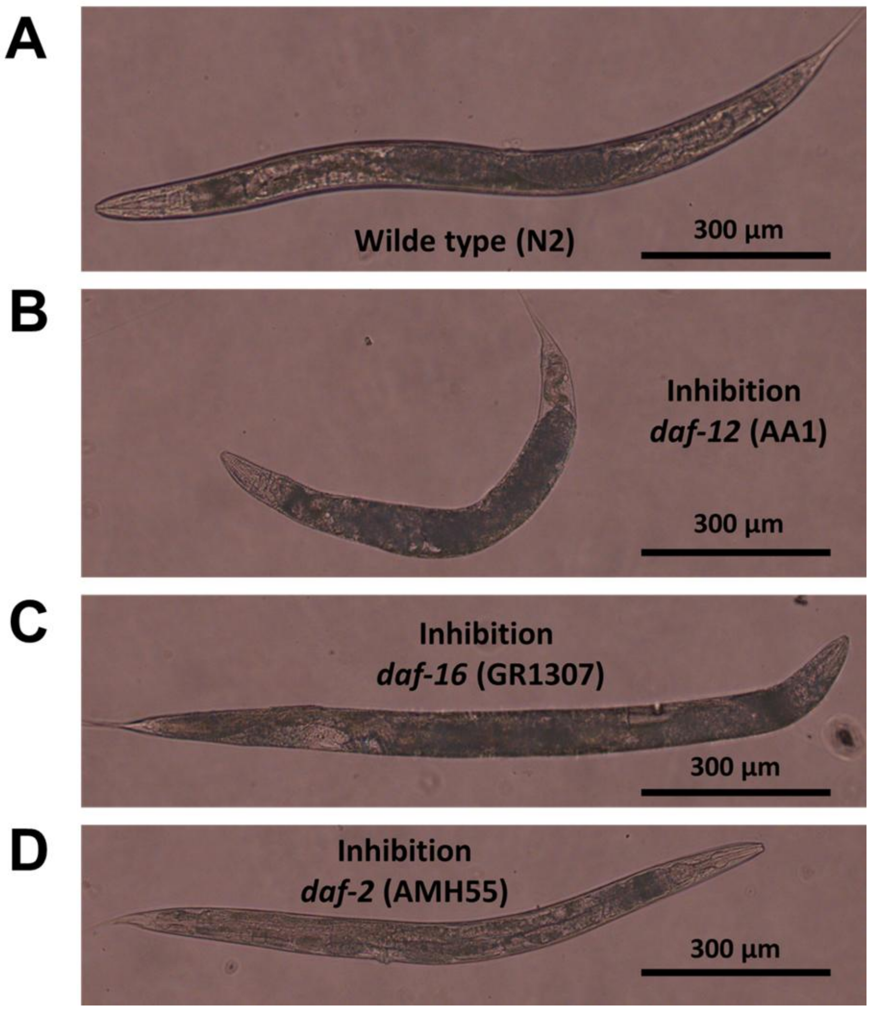

Fig. S11. Representative phenotype of adult-stage N2 and *daf* mutant. (**A**) wild-type N2, (**B**) *daf-12* mutant (AA1), (**C**) *daf-16* mutant (GR1307), and (**D**) daf-2 mutant (AMH55).

Fig. S12.

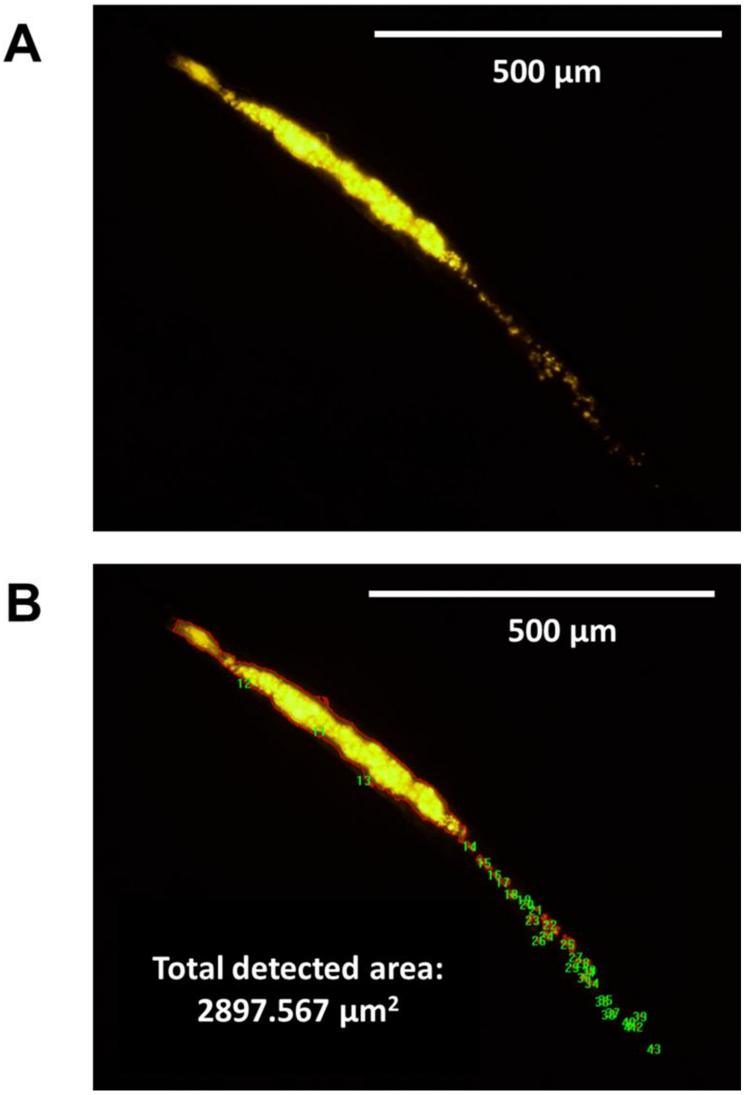

Fig. S12. Quantification of luminal Mi-PS under LPS exposure. (A) Representative fluorescence microscopy image of the AU37 worm showing luminally accumulated Mi-PS after exposure to LPS (100 µg/mL). The quantified animal was subjected to both the Mi-PS exposure period and the subsequent Mi-PS excretion period. (B) Example of fluorescence-area quantification using i-Solution software

Fig. S13.

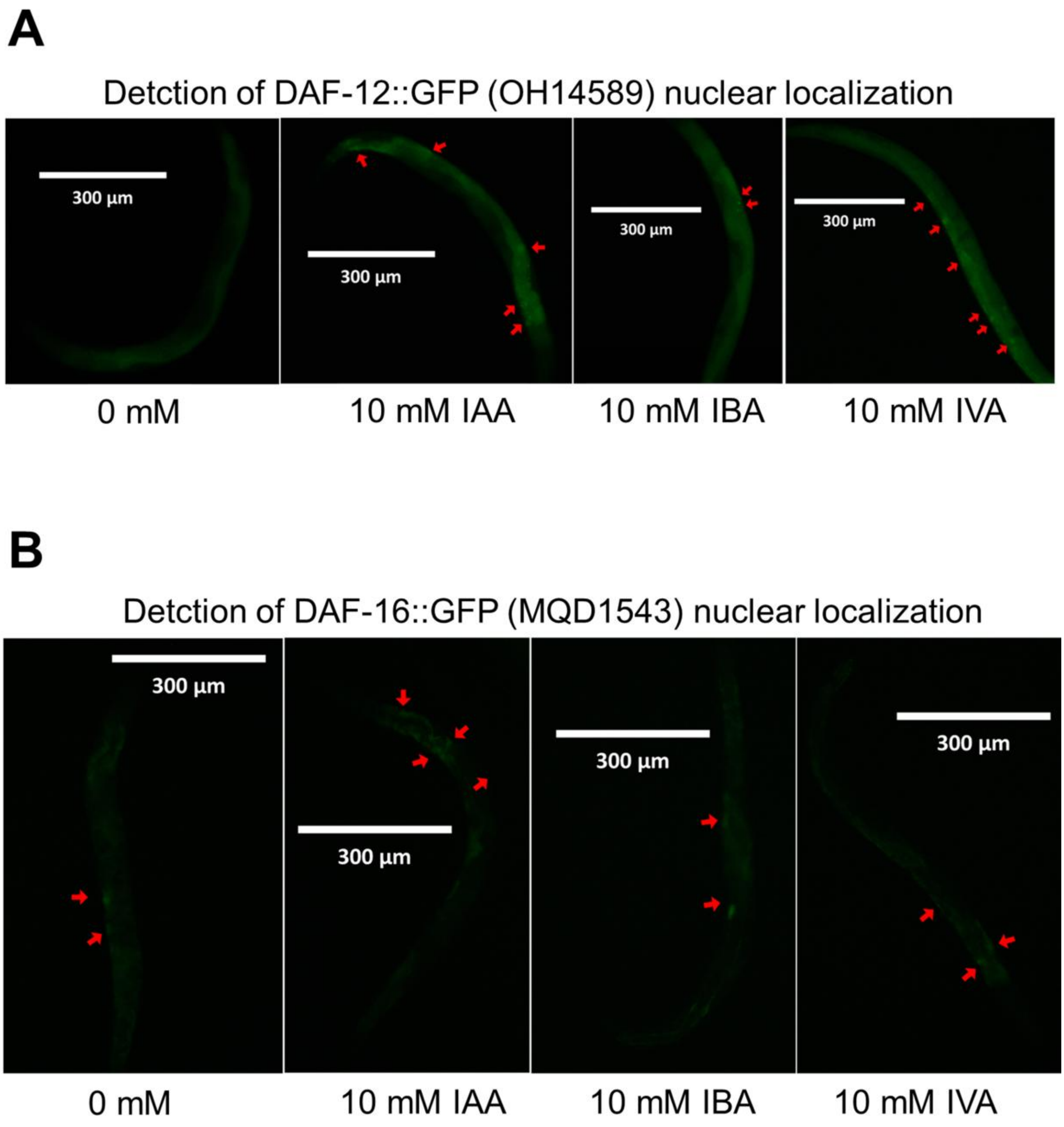

Fig. S13. Detection of DAF reporter nuclear localization after metabolite exposure. (A) Representative fluorescence images of DAF-12::GFP animals (OH14589) following 1-h exposure to isoamyl alcohol (IAA), isobutyrate (IBA), or isovalerate (IVA) at 10 mM compared with the 0 mM control. (B) Representative fluorescence images of DAF-16::GFP animals (MQD1543) under the same exposure conditions. Red arrows indicate nuclear puncta consistent with nuclear localization. Images were acquired using an Olympus IX53 microscope with i-Solution software under standardized acquisition settings.

Fig. S14.

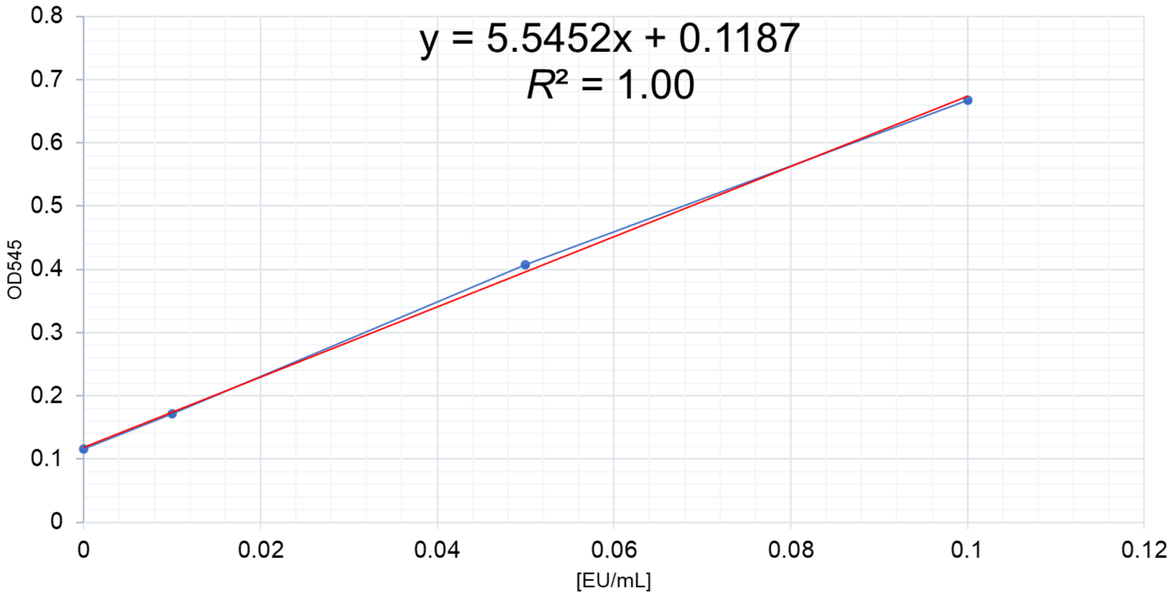

Fig. S14. Standard curve used for endotoxin quantification by the chromogenic LAL assay. Serial dilutions of endotoxin standards were measured at 545 nm, yielding a linear regression equation (y = 5.5452x + 0.1187) with $R^2 = 1.00$. This curve was applied to calculate endotoxin concentrations (EU/mL) in bacterial suspensions of *E. hormaechei* LG3 and *E. coli* OP50 incubated with or without Mi-PS.

Fig. S15.

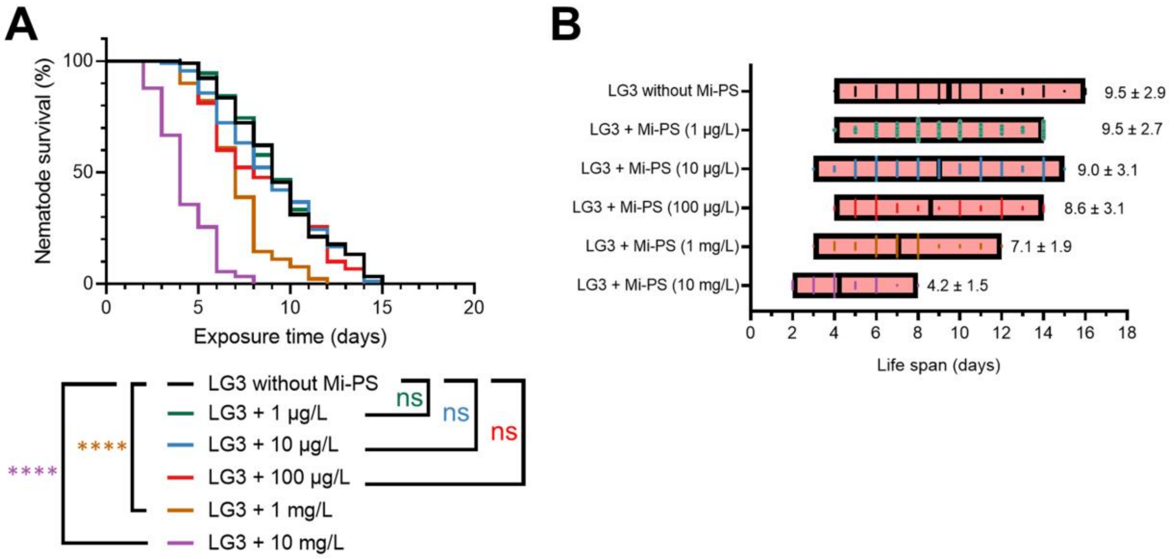

Fig. S15. Lifespan of *C. elegans* exposed to graded Mi-PS doses under LG3 feeding (**A**–**B**) To identify Mi-PS concentrations at which LG3–Mi-PS interactions elicit measurable lifespan effects in the host, we quantified *C. elegans* survival across Mi-PS doses (0–10 mg/L) on LG3 lawns. When Mi-PS was mixed into bacterial lawns at 0.03 g/mL at concentrations ≥ 1 mg/L, worms exhibited a significant reduction in lifespan compared with the control (LG3 without Mi-PS).

Fig. S16.

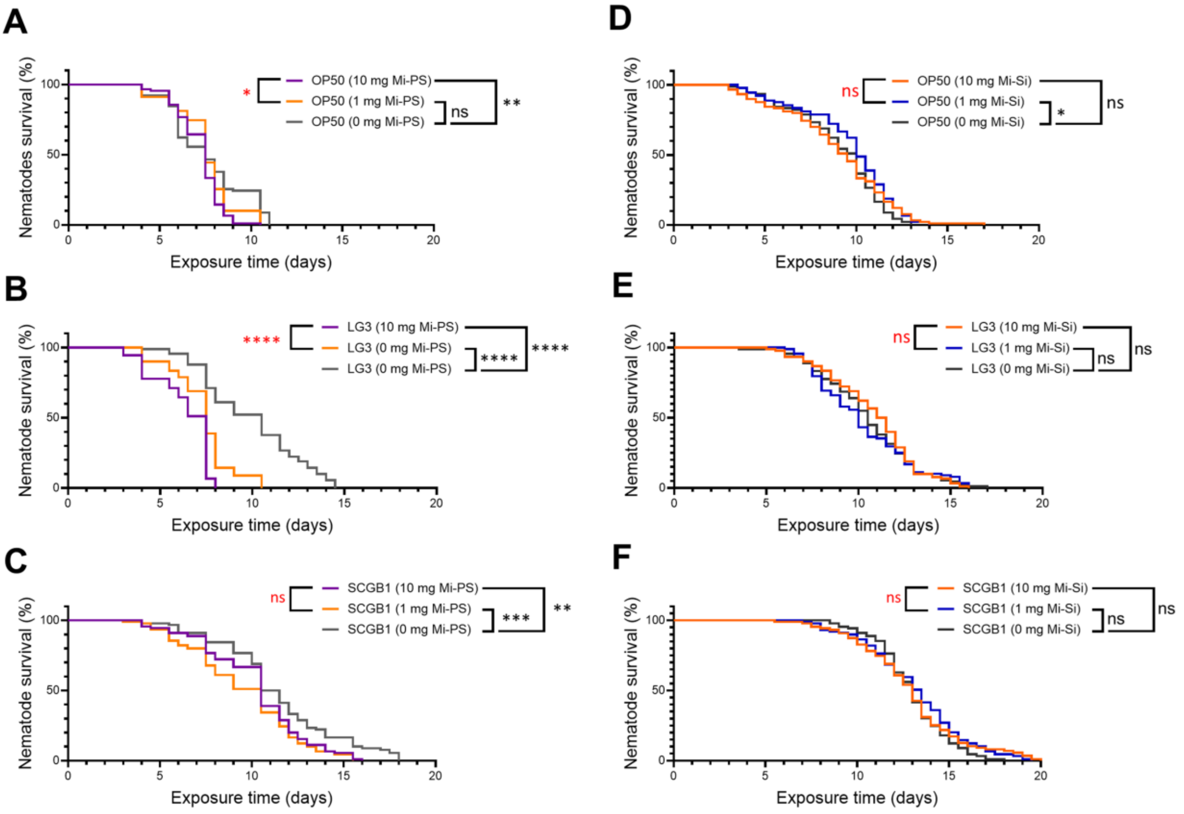

Fig. S16. Lifespan of *C. elegans* AU37 exposed to Mi-PS (0, 1, or 10 mg/L) or Mi-Si (0, 1, or 10 mg/L) under different bacterial diets. (**A**, **D**) Worms fed with *E. coli* OP50, (**B**, **E**) worms fed with *E. hormaechei* LG3, and (**C**, **F**) worms fed with *B. amyloliquefaciens* SCGB1. Survival assays were conducted under aerobic conditions at 25°C with bacterial biomass normalized to 0.03 g/mL. Each group contained 30 worms per plate, with experiments performed in triplicate.

Fig. S17.

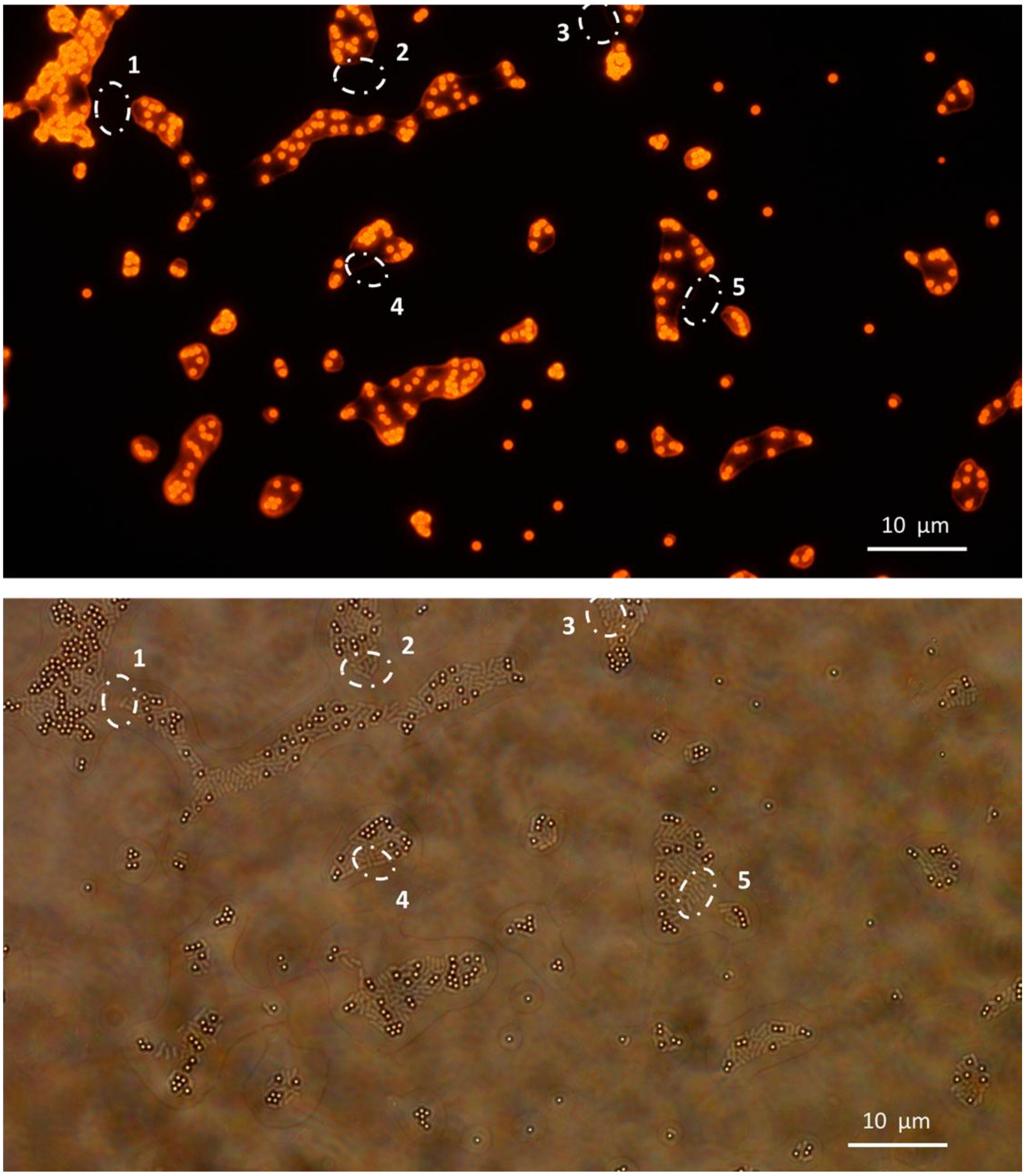

Fig. S17. Amorphous extracellular matrix developed around the Mi-PS. Representative paired micrographs of *E. hormaechei* LG3 biofilms incubated with fluorescent Mi-PS (top, fluorescence; bottom, bright-field). Dashed circles indicate regions where Mi-PS particles are not attached to the biofilm; in these regions, the amorphous extracellular matrix is not visible.

Fig. S18.

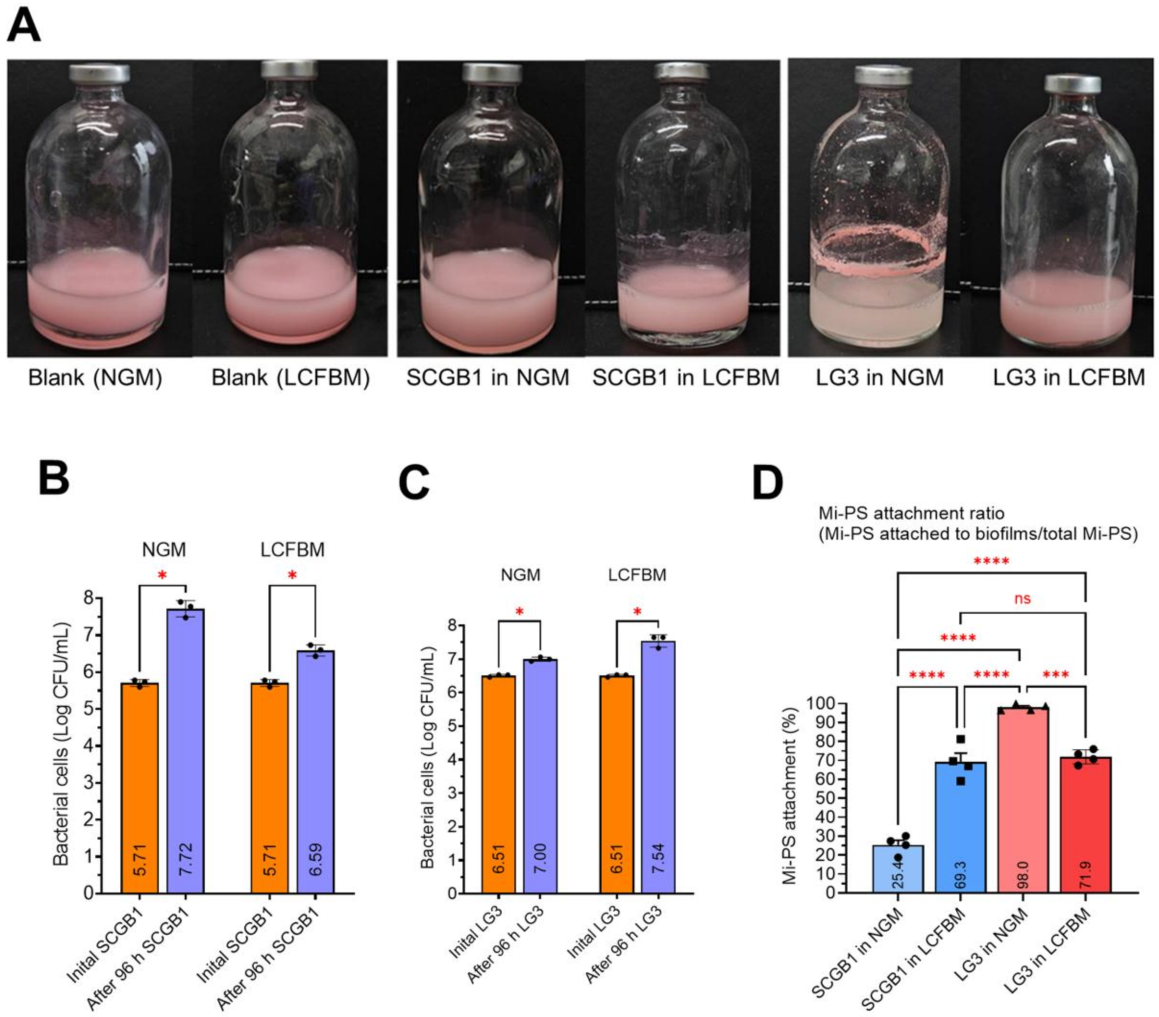

Fig. S18. Growth characteristics and Mi-PS aggregation differences of bacterial strains in liquid carbon-free basal medium (LCFBM). (**A**) Representative images of culture bottles after 96 h incubation of *B. amyloliquefaciens* SCGB1, and *E. hormaechei* LG3 in LCFBM and NGM liquid, compared with a blank control. Cultures were maintained under aerobic conditions at 180 rpm with headspace air refreshed every 48 h in the experimental room atmosphere. Strain-dependent differences in culture appearance and Mi-PS aggregation were clearly observed. (**B**–**C**) Changes in bacterial cell counts (log CFU/mL) before and after 96 h of incubation. For CFU enumeration, culture suspensions were vortexed for 10 s prior to serial dilution and plating. (**D**) For each culture, a 2-μL aliquot was placed on a microscope slide, and the Mi-PS attachment ratio was quantified from this 2-μL drop.

Fig. S19.

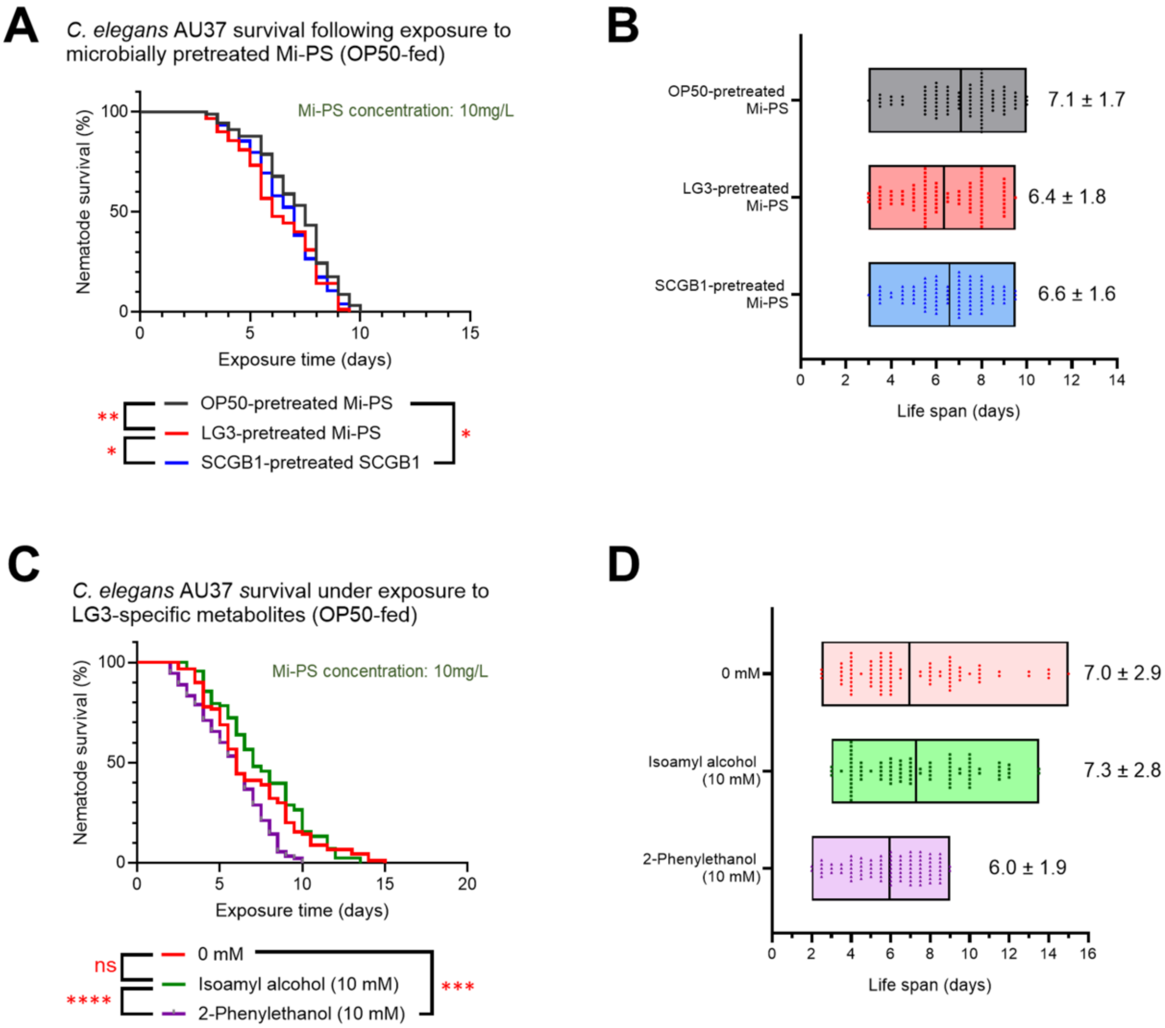

Fig. S19. Strain-specific effects of bacterial pretreated Mi-PS and volatile metabolites on AU37 lifespan. (**A**–**B**) Mi-PS was pretreated with each bacterial strain, recovered, and then re-exposed to OP50-fed worms (Mi-PS, 10 mg/L). (**C**–**D**) Lifespan effects of LG3-associated volatile metabolites on *C. elegans*. Isoamyl alcohol and 2-phenylethanol were supplemented at 10 mM during exposure (Mi-PS, 10 mg/L) in the OP50-fed background. These assays were conducted as part of the broader "lifespan analysis using biomass supplemented with volatile metabolites" experiment, in which isoamyl alcohol and 2-phenylethanol were evaluated alongside other strain-specific metabolites (e.g., isobutyrate and isovalerate) under the same experimental timeframe.

Fig. S20.

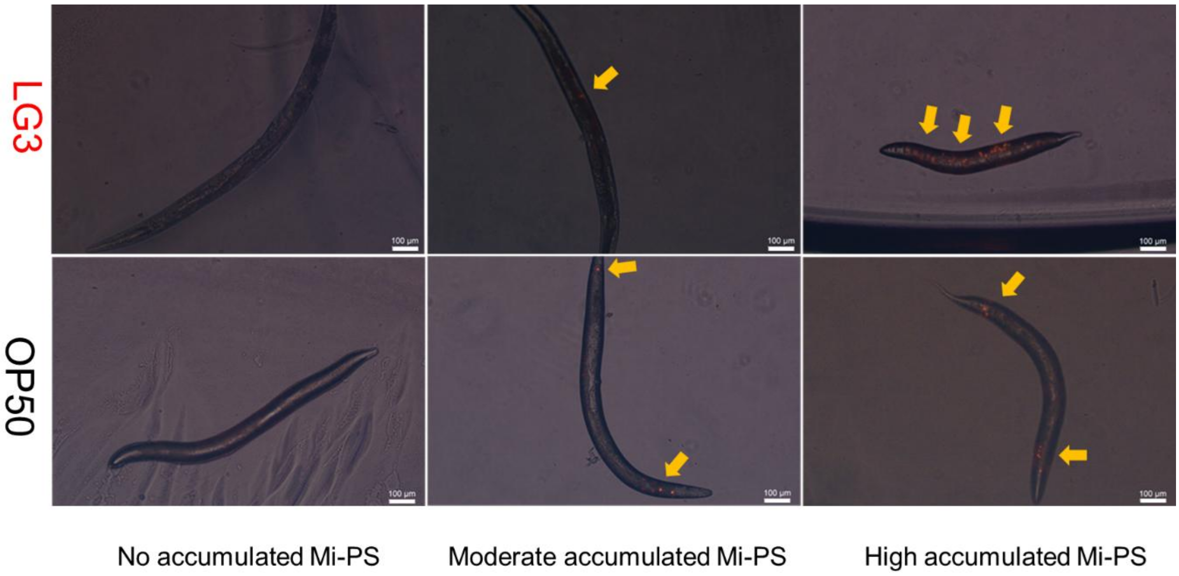

Fig. S20. *C. elegans* fed with LG3 or OP50 in the presence of 10 mg/L Mi-PS, showing morphological variation and differential levels of intestinal Mi-PS accumulation. Bright-field and fluorescence images were merged to visualize luminal Mi-PS particles (red signal). Worms are displayed with no accumulation (left), moderate accumulation (middle), or high accumulation (right).

Fig. S21.

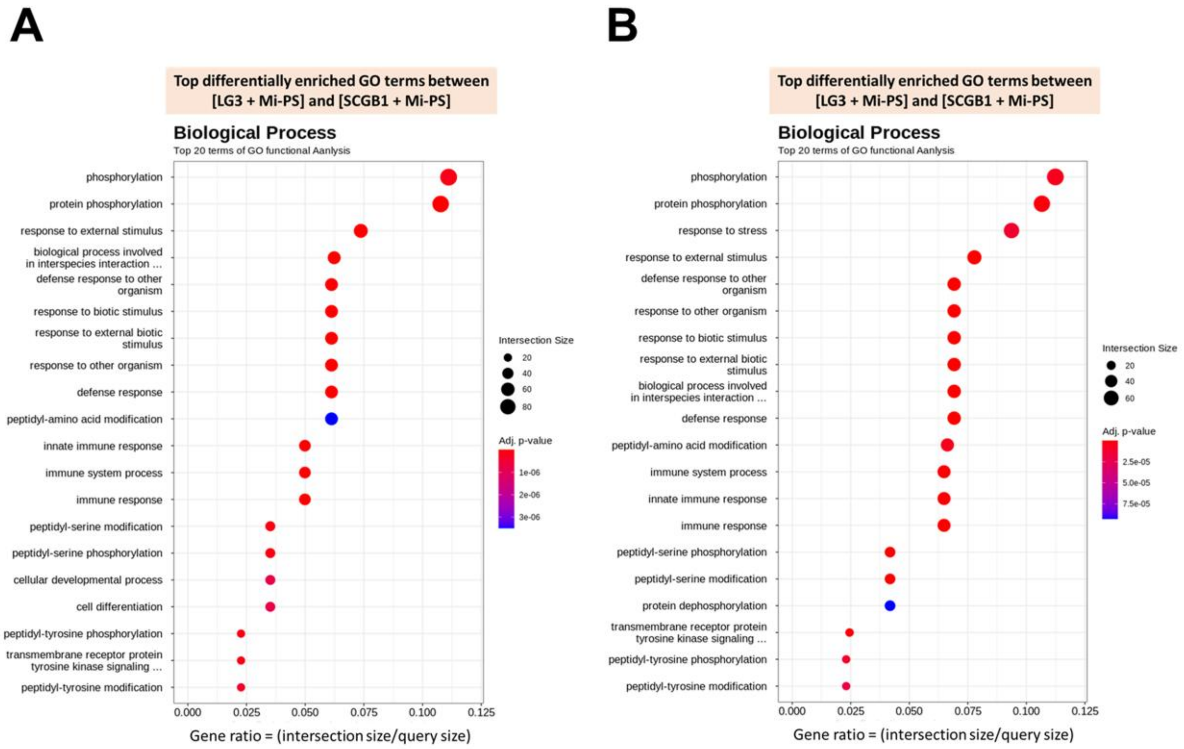

Fig. S21. Top differentially enriched GO terms in the Biological Process (BP) category from pairwise comparisons of worms exposed to Mi-PS (10 mg/L) under different bacterial feeding conditions. (**A**) Top 20 enriched BP terms from the LG3–SCGB1 comparison. (**B**) Top 20 enriched BP terms from the OP50–SCGB1 comparison. Within the BP category, phosphorylation and protein phosphorylation were significantly enriched, indicating differential regulation of signaling pathways in SCGB1-fed worms relative to LG3- and OP50-fed worms [31].

Fig. S22.

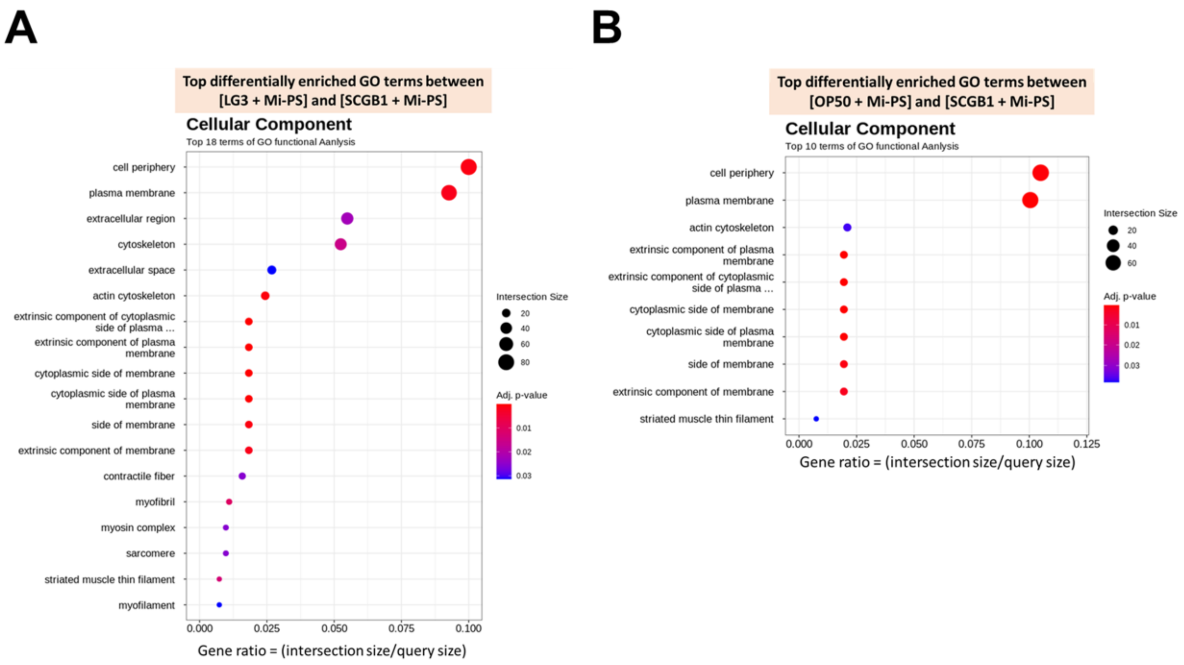

Fig. S22. Top differentially enriched GO terms in the Cellular Component (CC) category from pairwise comparisons of worms exposed to Mi-PS (10 mg/L) under different bacterial feeding conditions. (**A**) Comparison between *E. hormaechei* LG3– and *B. amyloliquefaciens* SCGB1–fed worms. (**B**) Comparison between *E. coli* OP50– and SCGB1–fed worms. In both comparisons, DEGs were significantly enriched in cell periphery, plasma membrane, and actin cytoskeleton, which represent key cellular sites of interaction with luminal Mi-PS [32]. LG3-fed worms exhibited additional enrichment in extracellular region and extracellular space, indicating stronger host–environment and host–microplastic interaction signatures compared with OP50-fed worms.

Fig. S23.

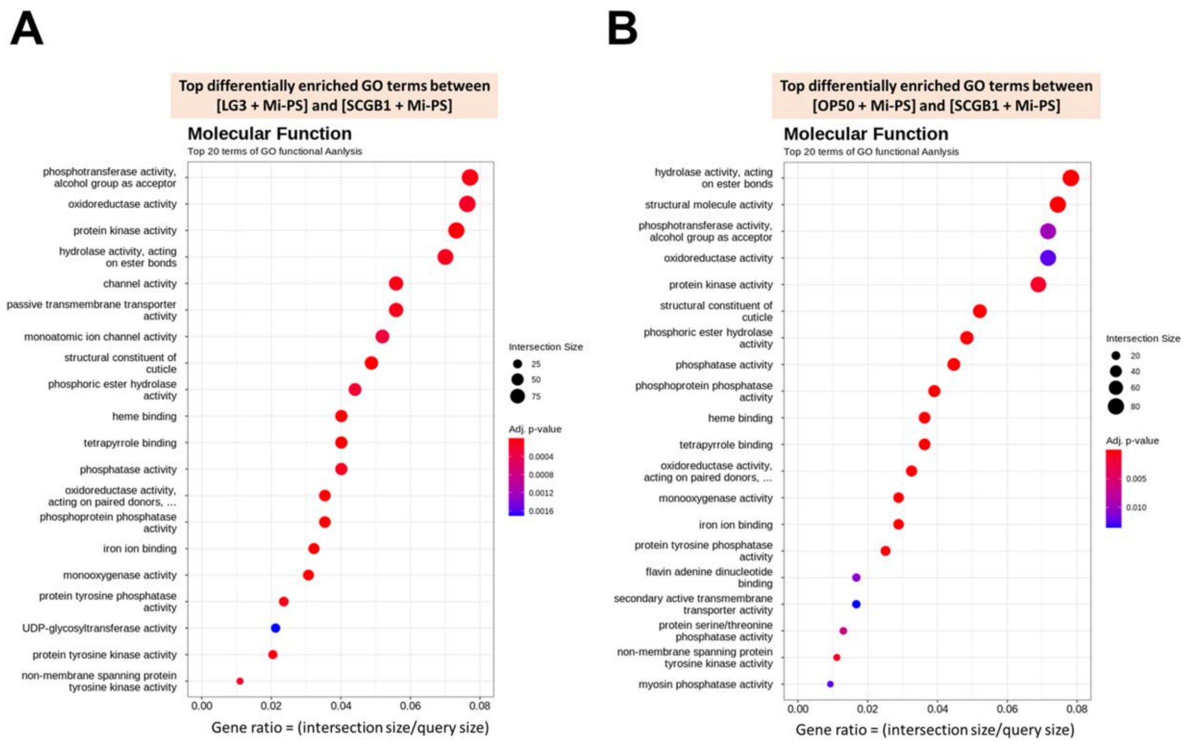

Fig. S23. Top differentially enriched GO terms in the Molecular Function (MF) category from pairwise comparisons of worms exposed to Mi-PS (10 mg/L) under different bacterial feeding conditions. (**A**) Comparison between worms fed *E. hormaechei* LG3 and *B. amyloliquefaciens* SCGB1. (**B**) Comparison between worms fed *E. coli* OP50 and SCGB1. Key enriched MF terms included oxidoreductase activity, protein kinase activity, phosphatase activity, and phosphotransferase activity, indicating that differences between feeding conditions were primarily driven by stress-related catalytic and signaling functions [33-35]. These patterns highlight that SCGB1-fed worms adopt a transcriptional program distinct from LG3- and OP50-fed worms, particularly in pathways linked to oxidative stress responses and signal regulation.

Fig. S24.

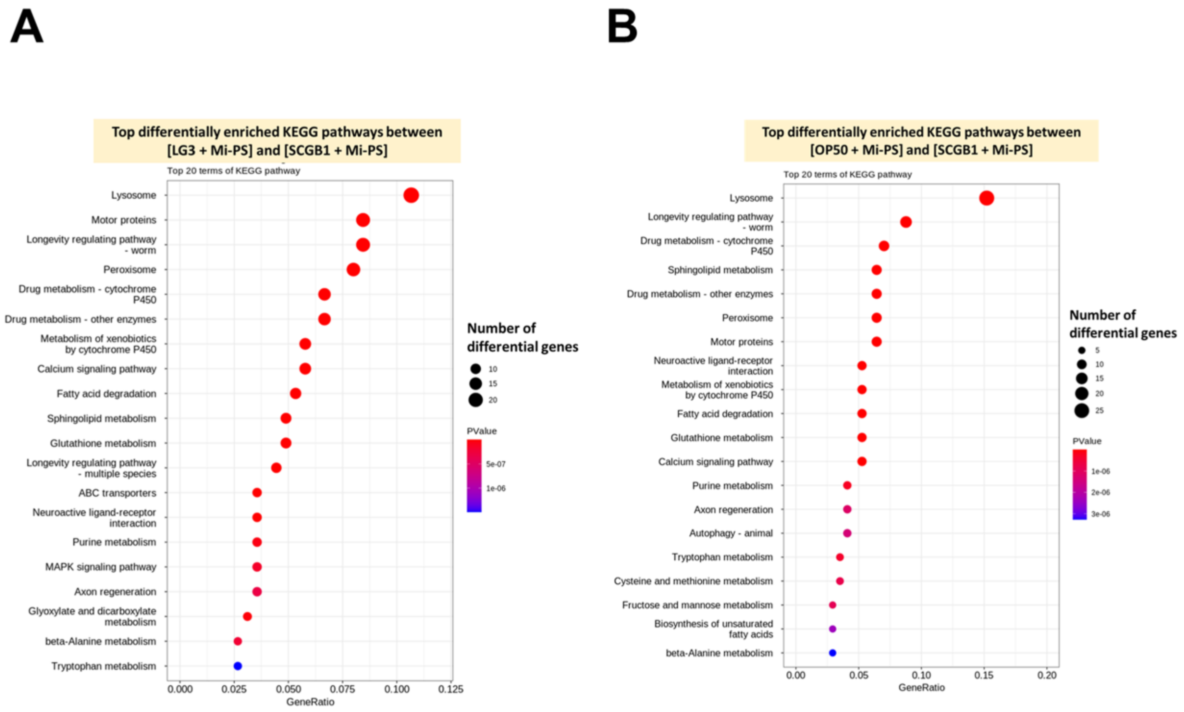

Fig. S24. KEGG pathway enrichment analysis of differentially expressed genes (DEGs) in *C. elegans* fed with distinct bacterial strains under Mi-PS (10 mg/L) exposure. (**A**) Top 20 enriched pathways from the LG3–SCGB1 comparison. (**B**) Top 20 enriched pathways from the OP50–SCGB1 comparison. Shared enrichments included lysosome, longevity regulating pathway, and cytochrome P450–related metabolism, suggesting broad differences in cellular homeostasis across feeding conditions [36]. LG3-fed worms exhibited additional enrichments in motor proteins, peroxisome, xenobiotic metabolism, and calcium signaling pathways.

Fig. S25.

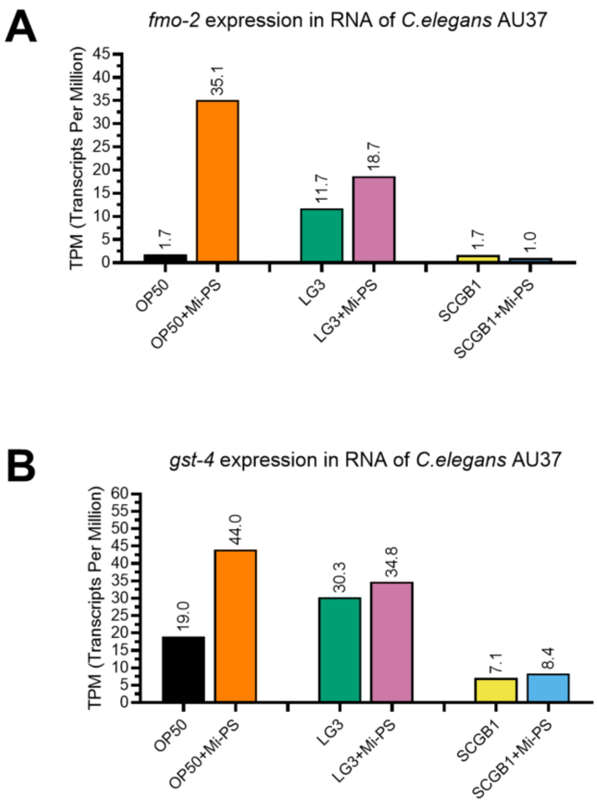

Fig. S25. Expression of oxidative stress reporters in *C. elegans* under Mi-PS exposure. (**A**) Expression of *fmo-2* and (**B**) expression of *gst-4* measured as TPM across worms fed with OP50, LG3, or SCGB1 in the absence or presence of Mi-PS (10 mg/L).

Supplementary video legends

Video S1. Differences in feeding behavior and pharyngeal activity among bacterial treatments. Representative feeding behavior of C. elegans on NGM plates after 100 h of exposure to Mi-PS and different bacterial strains.

Video S2. Microplastic ingestion and retention in the intestinal tract of *C. elegans* (**Video S2A**). Ingestion and excretion of Mi-PS by *C. elegans*. The video captures the passage of Mi-PS particles through the digestive tract under bacterial feeding conditions. (**Video S2B**). Luminally retained Mi-PS particles that are not excreted and remain stationary within the intestinal lumen. Time-lapse video recorded over 15 minutes and compressed to a 30-second playback (30× speed).

Video S3. Differences in locomotion behavior among bacterial treatments after Mi-PS exposure. Representative locomotion behavior of *C. elegans* on NGM plates after 100 h of exposure to Mi-PS and different bacterial strains.

Supplementary references:

1. Stiernagle T. Maintenance of *Caenorhabditis elegans*. WormBook. Pasadena, CA: WormBook, 2006;11:1-11.

2. Kang A, Choi HJ, Lee WJ *et al.* An integrated microbial genome-wide association studies-based characterization of nitric oxide homeostasis by probiotic *Bacillus subtilis* on aging and neurodegeneration using *Caenorhabditis elegans* and mouse models. *J Hazard Mater*. 2025;**495**:138902

3. Choi Y, Kwak M-J, Kang M-G *et al.* Molecular characterization and environmental impact of newly isolated lytic phage SLAM_phiST1N3 in the *Cornellvirus* genus for biocontrol of a multidrug-resistant *Salmonella* Typhimurium in the swine industry chain. *Sci Total Environ*. 2024;**922**:171208

4. Kang M-G, Kwak M-J, Kim Y. Polystyrene microplastics biodegradation by gut bacterial *Enterobacter hormaechei* from mealworms under anaerobic conditions: anaerobic oxidation and depolymerization. *J Hazard Mater*. 2023;**459**:132045

5. Chen S, Cheng J, Liu B *et al.* A novel *Bacillus velezensis* strain with the ability to simultaneously biodegrade polystyrene microplastics and fungicide carbendazim. *J Hazard Mater*. 2025;**495**:138860

6. Shang X, Lu J, Feng C *et al.* Microplastic (1 and 5 μm) exposure disturbs lifespan and intestine function in the nematode *Caenorhabditis elegans*. *Sci Total Environ*. 2020;**705**:135837

7. Wu X, Chen X, Jiang R *et al.* New insights into the photo-degraded polystyrene microplastic: effect on the release of volatile organic compounds. *J Hazard Mater*. 2022;**431**:128523

8. Karami A, Golieskardi A, Choo CK *et al.* A high-performance protocol for extraction of microplastics in fish. *Sci Total Environ*. 2017;**578**:485-94

9. Ainali NM, Kalaronis D, Kontogiannis A *et al.* Microplastics in the environment: Sampling, pretreatment, analysis and occurrence based on current and newly-exploited chromatographic approaches. *Sci Total Environ*. 2021;**794**:148725

10. Byerly L, Cassada R, Russell R. The life cycle of the nematode *Caenorhabditis elegans*: I. wild-type growth and reproduction. *Dev Biol*. 1976;**51**:23-33

11. Qi B, Kniazeva M, Han M. A vitamin-B2-sensing mechanism that regulates gut protease activity to impact animal's food behavior and growth. *Elife*. 2017;**6**:e26243

12. Kumar A, Saha MK, Kumar V *et al.* Heat-killed probiotic *Levilactobacillus brevis* MKAK9 and its exopolysaccharide promote longevity by modulating aging hallmarks and enhancing immune responses in *Caenorhabditis elegans*. *Immun Ageing*. 2024;**21**:52

13. Yu Y, Chen H, Hua X *et al.* Polystyrene microplastics (PS-MPs) toxicity induced oxidative stress and intestinal injury in nematode *Caenorhabditis elegans*. *Sci Total Environ*. 2020;**726**:138679

14. Uppaluri S, Weber SC, Brangwynne CP. Hierarchical size scaling during multicellular growth and development. *Cell Reports*. 2016;**17**:345-52

15. Bonnard E, Liu J, Zjacic N *et al.* Automatically tracking feeding behavior in populations of

foraging *C. elegans*. *Elife*. 2022;**11**:e77252

16. Sun J-D, Li Q, Haoyang W-W *et al.* Adsorption-based detoxification of endotoxins by porous flexible organic frameworks. *Mol Pharm*. 2022;**19**:953-62

17. Eliezer Y, Deshe N, Hoch L *et al.* A memory circuit for coping with impending adversity. *Curr Biol*. 2019;**29**:1573-83.e4

18. Edwards C, Canfield J, Copes N *et al.* D-beta-hydroxybutyrate extends lifespan in *C. elegans*. *Aging (Albany NY)*. 2014;**6**:621

19. Vannefors C. Detection and removal of endotoxin in nanomaterial preparations. Umeå, Sweden: Umeå University, 2022.

20. Schnaitman CA. Effect of ethylenediaminetetraacetic acid, Triton X-100, and lysozyme on the morphology and chemical composition of isolated cell walls of *Escherichia coli*. *J Bacteriol*. 1971;**108**:553-63

21. Cortés-López M, Gruner MR, Cooper DA *et al.* Global accumulation of circRNAs during aging in *Caenorhabditis elegans*. *BMC genomics*. 2018;**19**:8

22. Lee DJ, Eor JY, Kwak M-J *et al.* Enhanced Longevity and Immunity in *Caenorhabditis elegans* through ingestion of *Lactiplantibacillus plantarum* SKO-001: A Multi-Omics Study. *Food Sci Anim Resour*. 2024;**25**:1293-307

23. Robinson MD, Oshlack A. A scaling normalization method for differential expression analysis of RNA-seq data. *Genome Biol*. 2010;**11**:R25

24. Anders S, Huber W. Differential expression analysis for sequence count data. *Nat Prec*. 2010:1-1 https://doi.org/10.1038/npre.2010.4282.1

25. Benjamini Y, Hochberg Y. Controlling the false discovery rate: a practical and powerful approach to multiple testing. *J R Stat Soc Series B Stat Methodol*. 1995;**57**:289-300

26. Raudvere U, Kolberg L, Kuzmin I *et al.* g: Profiler: a web server for functional enrichment analysis and conversions of gene lists (2019 update). *Nucleic Acids Res*. 2019;**47**:W191-W98

27. Livak KJ, Schmittgen TD. Analysis of relative gene expression data using real-time quantitative PCR and the 2− ΔΔCT method. *methods*. 2001;**25**:402-08

28. Wagner OI, Esposito A, Köhler B *et al.* Synaptic scaffolding protein SYD-2 clusters and activates kinesin-3 UNC-104 in C. elegans. *Proceedings of the National Academy of Sciences*. 2009;**106**:19605-10

29. Ritz M, Garenaux A, Berge M *et al.* Determination of rpoA as the most suitable internal control to study stress response in *C. jejuni* by RT-qPCR and application to oxidative stress. *J Microbiol Methods*. 2009;**76**:196-200

30. Yang Y, Yang J, Wu W-M *et al.* Biodegradation and mineralization of polystyrene by plastic-eating mealworms: Part 1. Chemical and physical characterization and isotopic tests. *Environ Sci Technol*. 2015;**49**:12080-86

31. Burkard M, Betz A, Schirmer K *et al.* Common gene expression patterns in environmental model organisms exposed to engineered nanomaterials: A meta-analysis. *Environ Sci Technol*.

2019;**54**:335-44

32. Khan A, Jia Z. Recent insights into uptake, toxicity, and molecular targets of microplastics and nanoplastics relevant to human health impacts. *iScience*. 2023;**26**:106061

33. Gardon T, Morvan L, Huvet A *et al.* Microplastics induce dose-specific transcriptomic disruptions in energy metabolism and immunity of the pearl oyster Pinctada margaritifera. *Environ Pollut*. 2020;**266**:115180

34. Schieber M, Chandel NS. ROS function in redox signaling and oxidative stress. *Curr Biol*. 2014;**24**:R453-R62

35. Padmanabhan S, Mukhopadhyay A, Narasimhan SD *et al.* A PP2A regulatory subunit regulates *C. elegans* insulin/IGF-1 signaling by modulating AKT-1 phosphorylation. *Cell*. 2009;**136**:939-51

36. Tyshkovskiy A, Bozaykut P, Borodinova AA *et al.* Identification and application of gene expression signatures associated with lifespan extension. *Cell Metab*. 2019;**30**:573-93. e8

Thank you for your attention.
